# Differential gene expression drives cell-cycle-dependent transition from monopolar to bipolar growth in *Schizosaccharomyces pombe*

**DOI:** 10.1093/g3journal/jkag126

**Published:** 2026-05-11

**Authors:** Samridhi Pathak, Lauren M Tramonte, Maitreyi Das

**Affiliations:** Department of Biology, Boston College, Chestnut Hill, MA 02467, United States; Department of Biology, Boston College, Chestnut Hill, MA 02467, United States; Department of Biology, Boston College, Chestnut Hill, MA 02467, United States

**Keywords:** cell cycle, cell polarity, RNA seq, differential gene expression, stress, KEGG pathway

## Abstract

Cell polarity is important for maintaining cell structure and function. In *Schizosaccharomyces pombe*, after division, cells grow monopolarly from the old end, then transition to bipolar growth at a certain size when the new end activates. However, G1-arrested cells do not become bipolar despite continued growth, suggesting a role for the cell cycle in this process. To identify how the cell cycle impacts monopolar to bipolar transition, we performed high-throughput mRNA sequencing to detect differentially expressed genes in G1-arrested and G2-phase cells of the cell-cycle mutant *cdc10-129*. DESeq2 analysis identified 65 unique genes upregulated in G1 phase and 35 in G2 phase. Enrichment analysis shows that G1 phase cells upregulated the MAPK pheromone-response pathway, protein folding, rRNA processing, and heat-shock protein binding. G2 phase cells showed upregulation of plasma membrane maintenance and cell wall organization. In G2 phase cells, protein–protein interaction networks identified the *cdc15*-*hob3*-*rho1*-*bgs1* hub, known to promote bipolar growth and regulate cell wall biogenesis. In G1 phase cells, the *spk1-byr2-ste11* hub involved in pheromone-response and nutritional stress-dependent G1-arrest was identified. In agreement with these findings, we find that *spk1Δ* cells are precociously bipolar, indicating a role for this kinase in preventing bipolar growth. We hypothesize that bipolar growth in G2 cells requires a combination of factors that favor cell growth. In G2, stress response pathways are downregulated while anabolic pathways are upregulated, enabling transition from monopolar to bipolar growth.

## Introduction

Cells undergo polarization characterized by the asymmetric delivery and confinement of molecules and biological processes to establish and maintain cell shape (Srinivasan and Mishra 2009; Rappel and Edelstein-Keshet 2017; Quadri et al. 2020). Maintenance of cell shape is essential for tissue organization and proper function of the organism (Srinivasan and Mishra 2009). Cell polarity is a result of dynamic interactions of the plasma membrane with cytoskeletal proteins and the subsequent spatiotemporal organization of polarity complexes (Etienne-Manneville 2004; Chang and Martin 2009; Das and Verde 2013; Rappel and Edelstein-Keshet 2017). The molecular nature of the intrinsic and extrinsic spatial cues that establish structural and molecular asymmetry at the cell surface, although highly conserved, is poorly understood (Etienne-Manneville 2004; Bornens 2008; Chiou et al. 2021).

From yeast to humans, the key components that establish polarization, such as the Rho GTPases, are conserved, indicating that the underlying mechanisms have not significantly changed throughout evolution (Park and Bi 2007; Werner et al. 2011; Das and Verde 2013; Takeshita and Fischer 2019; Wolfenson et al. 2019; Pino et al. 2021). The fission yeast, *Schizosaccharomyces pombe* cells offer a simpler bipolar model of cell polarization regulated via the small GTPases Cdc42, a major regulator of cell polarity (Arellano et al. 1999a; Perez and Rincon 2010; Martin and Arkowitz 2014; Pino et al. 2021; Rutkowski et al. 2024). Cdc42 is activated by GEFs (guanine nucleotide exchange factors) and inactivated by GAPs (GTPase-activating proteins) (Etienne-Manneville 2004; Rincon et al. 2007; Das et al. 2012; Das and Verde 2013; Pino et al. 2021; Rutkowski et al. 2024).

In fission yeast, the cell polarity pattern is cell-cycle-dependent. Post-cell division, cells are monopolar and initiate growth from the old end that existed in the previous generation. The new end, which is generated during cell division, initiates growth, resulting in bipolar cells after a process called new-end-take-off (NETO). However, what drives NETO during bipolar growth is poorly understood (Das et al. 2012; Das and Verde 2013). Previous models suggest that bipolarity occurs once the cell achieves a certain cell size during growth in the G2 phase (Das et al. 2012; Das and Verde 2013).

In the G2 phase, GTP-bound Cdc42 oscillates between the 2 ends in an anticorrelated manner, promoting bipolar growth (Das et al. 2012; Das and Verde 2013; Hercyk et al. 2019; Hercyk and Das 2019; Harrell et al. 2024). The anticorrelated oscillations are due to the competition between the 2 growing ends for Cdc42 activation (Das et al. 2012; Das and Verde 2013; Harrell et al. 2024). As the cells increase in size and synthesize sufficient proteins, the competition between the ends is reduced (Das et al. 2012; Chiou et al. 2018, 2021; Crocker et al. 2025). This suggests that as the cell size increases, bipolar Cdc42 activation at the cell ends is efficiently established, allowing simultaneous growth at these sites. A second model defines that Gef1, a secondary activator of Cdc42, acts as a spark-plug to initiate the Cdc42 positive feedback at the new end to allow bipolar growth (Hercyk et al. 2019; Hercyk and Das 2019).

It has also been shown that the transition to bipolar growth is cell-cycle-dependent. Cell-cycle mutants arrested in the G1 phase show increased cell size but remain monopolar (Stern and Nurse 1997). This indicates that bipolarity occurs in the G2 phase, and that cell size increase alone does not explain the transition from monopolar to bipolar growth. Cdc10 protein is a DNA-binding transcription factor responsible for the transcription of genes such as *cdt2*, *cig2*, cdc18, *mik1*, *nrm1*, *rec11*, *rec8*, and *yox1* (Nakashima et al. 1995; Baum et al. 1997). In the *cdc10-129* temperature-sensitive mutant, the cells grow in length but remain monopolar under restrictive conditions (Nakashima et al. 1995; Baum et al. 1997; Aligianni et al. 2009).

Research in cell polarization mainly examines the Rho GTPase signaling pathways and their downstream effects at the polarized sites. Changes in the regulation of the signaling pathways result in phenotypes such as loss of polarity or monopolarity with corresponding changes in cell dimensions (Das et al. 2007, 2009, 2012, 2015; Kelly and Nurse 2011; Hercyk and Das 2019; Pino et al. 2021; Harrell et al. 2024). However, the growing ends of the monopolar *cdc10-129* mutant do not show any major differences in the regulation of Cdc42 or cellular dimensions. This suggests that the monopolarity defect observed in these cells could be due to a defect in the global regulation of polarity. We asked if the transition from monopolar to bipolar growth correlates with the differences in gene expression in the different cell-cycle stages. Indeed, gene expression data from different organisms suggest that cell polarity is also regulated at the systems level. Polarity genes show divergent expression profiles in distinct human tissues and cancer cells (Lin et al. 2015). *Drosophila* embryos exhibit cell-cycle-dependent changes in polarity gene expression (Payankaulam et al. 2022). Nevertheless, it remains unclear how changes in gene expression result in different polarization states.

Here, we used high-throughput mRNA sequencing for gene expression analysis using the *cdc10-129* mutant to identify cell-cycle-dependent factors that correlate with the transition from monopolar to bipolar growth. We compared the gene expression profiles of cells in the G1 and G2 phases using the DESeq2 R package. Gene enrichment analyses and protein–protein interaction (PPI) network indicate that cells in the G1 phase of the cell cycle show upregulation of specific stress response pathway genes involved in G1-arrest. On the other hand, the G2 phase shows upregulation of genes involved in plasma membrane modulation and cell wall biosynthesis. We further evaluated the cell-cycle-dependent expression patterns of these genes via qPCR. In agreement with our gene expression analyses, we find that the stress kinase *spk1*, upregulated in G1, prevents precocious bipolar growth, while the genes *cdc15* and *hob3*, upregulated in G2, prevent monopolar growth. Our data indicate that nutritional stress response/G1-arrest pathways are upregulated in the G1 phase. In the absence of nutritional stress, these pathways are downregulated to not only promote cell-cycle progression but also promote bipolar growth. In the G2 phase, the pathways that promote cellular growth, including plasma membrane maintenance and cell wall organization, are upregulated. Our data indicate that cell-cycle phase-dependent differential gene expression promotes the transition from monopolar to bipolar growth.

## Materials and methods

### Strains and genetic methods

All *S. pombe* strains used in this study are listed in Supplementary Table 1. The strains used in the study were isogenic to the original strain PN567. The cells were grown in YES (yeast extract, Sunrise Science) media at 25 °C unless otherwise specified. Standard yeast genetic manipulation techniques were used for cell isolation, growth, and analysis (Moreno et al. 1991). Cells were cultured to mid-log phase for 3 consecutive days, where they were diluted every 8 generations.

### Cell-cycle progression and synchronization analysis for *cdc10-129* mutant cells

The cell-cycle block-and-release experiment was performed for strains carrying the *cdc10-129* allele and control wild-type cells (Tormos-Perez et al. 2016). Early log phase cells were shifted from 25 to 36 °C for 4 h, then shifted back to 25 °C for the indicated times. Cell synchronization of the *cdc10-129* mutant was observed every 30 min post-release at 25 °C through calcofluor and DAPI staining. Microscopic imaging was performed on a Nikon Eclipse Ti2 microscope with a 60× objective to determine the percentage of cells with septa and the percentage of bi-nucleated cells. Calcofluor and DAPI staining was completed for 3 independent replicates.

### Cell-cycle synchronization, sample collection, and RNA isolation for high-throughput mRNA sequencing

The experimental design for sample collection is illustrated in Supplementary Fig. 1a. In brief, early log phase *cdc10-129* mutant and control wild-type cells were grown at 36 °C for 4 h, followed by release at 25 °C for 150 min. Cells were collected from samples grown at 36 °C and at 25 °C for 150 min for RNA isolation. The asynchronous controls grown at 25 °C were also collected for RNA isolation. All 6 different cell types collected were then subjected to centrifugation at 3,750 rpm for 10 min at 4 °C. The supernatant was discarded, while the cell pellet was further used for RNA isolation. RNA isolation was done using the RNeasy Mini Kit from Qiagen (Cat No. 74104) with the standard kit protocol.

### Library construction, quality control, and sequencing

The project workflow is summarized in Supplementary Fig. 1b. Messenger RNA was extracted from total RNA using poly-T oligo-attached magnetic beads. Following fragmentation, the first strand cDNA was synthesized with random hexamer primers, followed by the second strand cDNA synthesis with either dUTP for a directed library or dTTP for a nondirectional library. The nondirectional library was prepared following end repair, A-tailing, adaptor ligation, size selection, amplification, and purification as shown in Supplementary Fig. 2a. The directional library was prepared following end repair, A-tailing, adapter ligation, size selection, USER enzyme digestion, amplification, and purification, as shown in Supplementary Fig. 2b. The library was quantified using Qubit and real-time PCR, and its size distribution was detected using a bioanalyzer. Libraries were sequenced on an Illumina Novaseq 6000, S4 flow cell using a paired-end 150 (PE150) workflow. We targeted a total of 20 M paired reads per sample (6 Gb raw data per sample). Based on the wide-ranging evaluation parameters for RNA sample quality requirements for library preparation and sequencing, all 18 samples passed the quality control measures for further analysis and are reported in Supplementary Table 2.

### Bioinformatic analysis pipeline and data quality control

The workflow for mRNA sequencing data of standard bioinformatic analysis with a well-annotated reference genome is shown in Supplementary Fig. 3a. We performed a variety of bioinformatic analyses, as shown in Supplementary Fig. 3b, to evaluate the differential gene expression.

CASAVA base recognition (Base Calling) was used to transform the original image data file from the high-throughput sequencing platform, Illumina. Raw data were then stored in FASTQ (fq) format files, which comprised read sequences and base quality values. Raw data (raw readings) were initially processed using Perl scripts. The step, as shown in Supplementary Fig. 4a, produced clean data (clean reads) by removing adaptors, poly-N, and low-quality reads from raw data. We also computed the Q20, Q30, and GC contents. The adaptor sequence used in the current pipeline is summarized in Supplementary Fig. 4b. The sequencing error rate for each base was calculated by the Phred score, represented by the equation below,


Qphred=-10log10(e),

where “e” stands for sequencing error rate, and Qphred represents base quality values obtained from Illumina platforms. The parameters for computing single-base error rates are provided in Supplementary Table 3, and the data are provided in Supplementary Table 4.

Raw sequencing data and processed count matrices are available in NCBI GEO under accession number GSE326505.

### Mapping sequencing reads to the reference genome

The PomBase genome website was the source for the reference genome and gene model annotation files (Rutherford et al. 2024). The reference genome index was constructed, and paired-end clean reads were matched to the reference genome using the graph-based alignment tool Hisat2 v2.0.5.

### Novel transcript predictions

The mapped reads of each sample were assembled by StringTie (v1.3.3b) (Pertea et al. 2015) in a reference-based approach. StringTie uses a novel network flow algorithm as well as an optional de novo assembly step to assemble and quantify full-length transcripts representing multiple splice variants for each gene locus.

### Quantification of gene expression levels

The number of reads mapped to each gene was counted using featureCounts v1.5.0-p3 (Liao et al. 2014). The length of the gene and the number of reads mapped to it were then used to compute the FPKM (fragments per kilobase of transcript per million) of each gene. Upon quantification of gene expression, statistical analysis for the expression data was performed to screen for the differentially expressed genes in different conditions. The differential analysis was mainly divided into 3 steps. First, the raw read count was normalized to correct the sequencing depth, followed by applying the statistical model and calculating the hypothesis test's probability or *P*-value. Finally, we utilized multiple hypothesis test corrections to obtain FDR values (false discovery rate) (Anders and Huber 2010). For different experimental conditions, we selected appropriate software for gene expression differential analysis, as shown in Supplementary Table 5.

### Differential expression of gene analysis

Differential expression (Anders and Huber 2010) analysis of 2 conditions/groups (3 biological replicates per condition) was performed using the DESeq2R package (1.20.0) (Love et al. 2014). For each sequenced library, the read counts were adjusted by the edgeR program package (Robinson et al. 2010) through one scaling factor. edgeR R package (3.22.5) was used to analyze the differential expression between 2 conditions. Benjamini & Hochberg method was used to adjust the *P*-values. An absolute fold change of 2 and a corrected *P*-value of ≦0.05 were set as the threshold for significant differential expression of genes.

### GO and KEGG enrichment analysis of differentially expressed genes

The clusterProfiler R package with gene length bias correction was used for Gene Ontology (GO) (Young et al. 2010) enrichment analysis of differentially expressed genes. GO terms with a corrected *P*-value ≦0.05 were considered significantly enriched by differentially expressed genes. We used the clusterProfiler R package to test the statistical enrichment of differential expression genes in the Kyoto encyclopedia of genes and genomes pathways (KEGG) (Kanehisa and Goto 2000).

### Gene set enrichment analysis

Gene set enrichment analysis (GSEA) (Mootha et al. 2003; Subramanian et al. 2005) was used to determine if a predefined Gene Set showed any significant consistent difference between 2 biological states. All the genes were ranked according to the degree of differential expression in the 2 samples. The predefined gene sets were analyzed to ensure the reliability of enrichment at the top or the bottom of the gene list. Subtle gene expression changes can be analyzed using the GSEA analysis. We used GO and KEGG datasets independently for GSEA analysis (http://www.broadinstitute.org/gsea/index.jsp).

### SNP analysis

GATK (v4.1.1.0) (McKenna et al. 2010) software was used to perform SNP calling. Raw VCF files were filtered with GATK standard filter method together with other parameters (cluster:3; WindowSize:35; QD < 2.0; FS > 30.0; DP < 10. 2.9).

### AS analysis

We used rMATS (4.1.0) (Shen et al. 2014) software to analyze the Alternative splicing event.

### PPI analysis of differentially expressed genes

We used the STRING database (Szklarczyk et al. 2019) with known and predicted PPI to perform PPI analysis of differentially expressed genes.

### Cell morphological analysis of the *spk1Δ* mutant

Early log phase cells of control wild-type and *spk1Δ* cells growing at 25 °C were imaged using calcofluor staining of the cell wall on the Nikon Eclipse Ti2 microscope with a 60× objective. The morphological analysis of percentage monopolar and bipolar cells was calculated using the cell counter feature on ImageJ/Fiji software.

### RNA isolation, cDNA synthesis, and qPCR gene expression analysis of wild-type, *cdc10-129*, and *cdc25-22* mutant cells

mRNA was extracted from wild-type and *cdc10-129* mutant strains grown at 25 °C, 36 °C for 4 h and then shifted to 25 °C for 150 min, using Qiagen RNAeasy Mini kit (Cat. No. 74104). The isolated mRNA was then used to synthesize stable cDNA using NEB LunaScript RT SuperMix Kit (E3010). Real-time quantitative PCR was performed using the NEB Luna Universal One-Step RT-qPCR kit. PCR primers for each of the genes were ordered from IDT (Integrated DNA Technology) and are reported in Supplementary Table 6. The primer efficiency for all the genes, including the housekeeping gene and the gene of interest, was determined by calculating absolute target quantities from an appropriate standard curve, which was derived for each gene from a series of known dilutions. The primer efficiency for each gene was calculated from the standard curve using the equation y=mx+c with an *R*^2^ value of 0.98.

We used dye-based real-time fluorescence of a double-stranded DNA (dsDNA) binding dye, SYBR Green I, to measure DNA amplification after each PCR cycle. Upon fluorescence signal detection over the background fluorescence, a quantification cycle or Ct/Cq values were determined on the QuantStudio Real-Time PCR machine (v1.7.2). Ct/Cq values were estimated from 3 independent biological replicates, which were then used to evaluate the relative target abundance between 2 or more samples. The expression levels for *hob3*, *cdc15*, and *spk1* were normalized against *act1* housekeeping gene using a robust method (Pabinger et al. 2014; Ganger et al. 2017). The equation used for log fold change determination is


$$logfold\;change=2^{\wedge }(\Delta \Delta Ct)$$

Data were presented as mean ± s.d. Statistical significance of fold change between 2 conditions was determined using the paired Student's *t*-test.

## Results

### Cell-cycle-dependent RNA isolation in *cdc10-129* mutants for gene expression analysis

Cell-cycle progression of *cdc10-129* mutant cells was performed using canonical cell-cycle arrest and release experiments. *cdc10-129* mutant cells growing in early log phase at 25 °C were arrested in the G1 phase at 36 °C for 4 h. The cells were then released to 25 °C and monitored for cell-cycle progression using DAPI and calcofluor staining. We observed that cells arrested at 36 °C are monopolar (Supplementary Fig. 5a). When released to 25 °C, more than 90% of the cells are bipolar after 2 h and 30 min (Supplementary Fig. 5b), and bi-nucleate after 3 h, suggesting entry into mitosis. This indicates that 2 h and 30 min after release, the cells are synchronized in G2 phase.

The mRNA experimental design workflow for cell synchronization using *cdc10-129* mutants is shown in Supplementary Fig. 1a. Early log phase cells growing at 25 °C (asynchronous), arrested at 36 °C (G1), and post-release at 25 °C (G2) for 2 h and 30 min were collected for RNA isolation. Similar conditional controls with wild-type cells were also collected for RNA extraction and high-throughput sequencing. The isolated RNA was used for library construction, quality control, and sequencing as per the workflow shown in Supplementary Fig. 2a and b. We next analyzed the quality of the RNA sequencing data by estimating the error rate distribution, GC content distribution, and data filtering. In our quality control analysis, we found that the single-base error rate for all 18 samples was lower than 1% (Supplementary Table 4). Analysis of GC content distribution showed equal and stable G-C and A-T content throughout the sequencing process for all 18 samples (Supplementary Table 4).

### Differential expression of gene analysis shows cell-cycle-dependent differences in gene regulation

The gene expression data were statistically analyzed using the DESeq2 R package between the different groups. Variability between biological replicates was calculated using edgeR to determine the variability within biological replicates. Post-statistical analysis, the alignment of the sequence was performed using HISAT2 with the reference genome (Supplementary Table 7). Mapped regions are classed as exons, introns, or intergenic regions. The distribution of sequencing reads across all samples in the genomic area was found to have 97% to 99.5% exons, and the remaining area belonged to either introns or intergenic regions. The gene expression data for all 18 samples from 3 independent samples are summarized in Supplementary Table 8. Distribution of gene expression levels under different conditions and fragments per kilobase per million mapped fragments (FPKM) among different samples is represented as boxplots shown in Supplementary Fig. 4c. For biological replicates, the final FPKM is represented as the mean value. The comparison for all samples is provided in Supplementary Table 9.

We used Pearson correlation coefficient to calculate the differential expression between the samples. A correlation coefficient closer to 1 indicates high similarity within the samples. As per ENCODE (ENCyclopedia Of DNA Elements) (ENCODE Project Consortium 2004), the square of the Pearson correlation coefficient should be greater than 0.92, and the *R*^2^ value should be greater than 0.8. We calculated the inter-sample correlation coefficient *R*^2^ value within the range of 0.8 to 1 (Supplementary Fig. 6a).

**Fig. 1.**
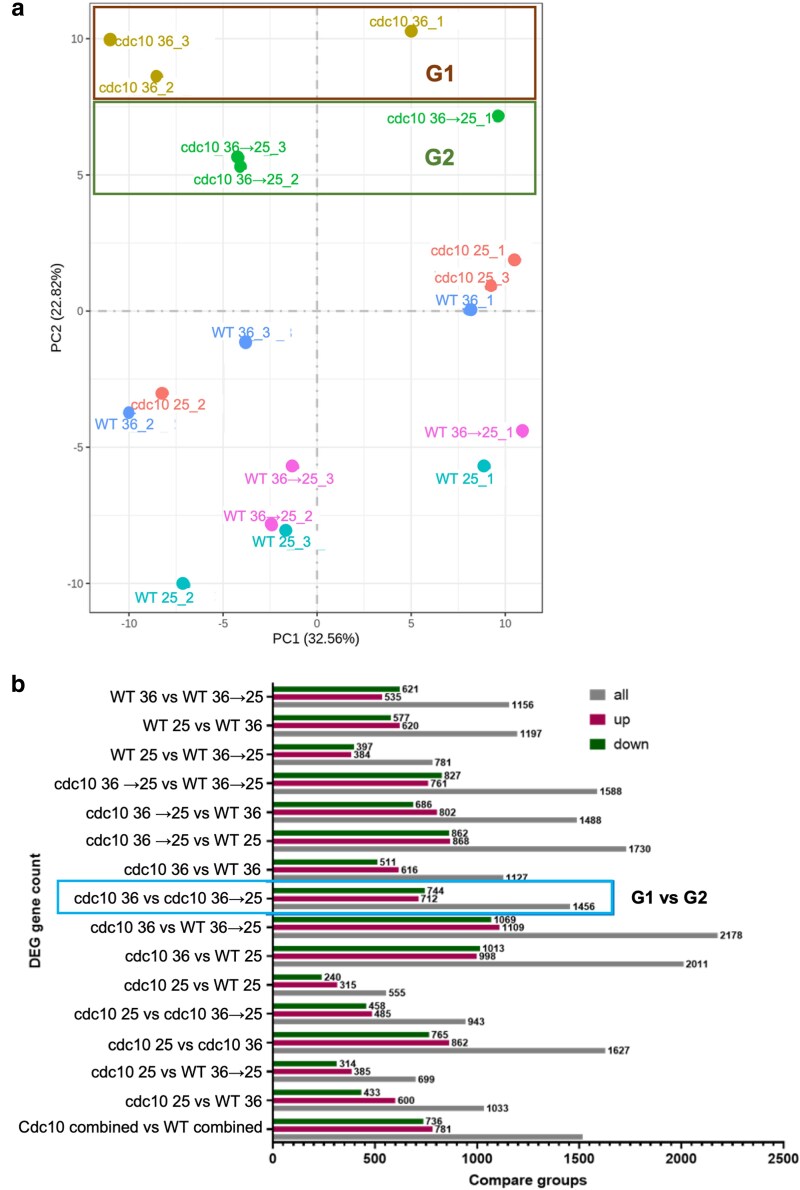
Quality control of high-throughput mRNA sequencing data. a) PCA result representing intergroup differences and intragroup sample duplication. G1 and G2 phase samples are highlighted in individual boxes. b) Histogram for the number of differential genes for each comparison combination. For each sample the top bar depicts downregulated genes, middle bar depicts upregulated and bottom bar depicts total differetailly regulated genes, and the numbers on the columns indicate the differential genes.

To evaluate the intergroup differences and intragroup sample duplication, we utilized the principal component analysis (PCA) method. We performed PCA analysis on the gene expression value (FPKM) of all samples (Fig. 1a). We find a distinct clustering pattern between *cdc10-129* cells growing in G1 and G2 phase of the cell cycle, with principal components 1 and 2 (PC1 and PC2) accounting for 32.56% and 22.82% of the total variance, respectively. While the G1 and G2 phase cells showed distinct clusters, one biological replicate in each group did not cluster as tightly, indicating some degree of biological variability within these groups. All other conditional controls from wild-type cells and the early log phase *cdc10-129* cells that were asynchronous in different cell-cycle stages were widely dispersed. PC1 captures the dominant source of variance across all samples, which in our dataset is the broad transcriptional variability present in unsynchronized cells grown under different temperature conditions, reflecting variation in cell-cycle-stage distribution, metabolic state, and temperature response. This large, heterogeneous variance dominates PC1, and since it is not specific to any one experimental replicate group, replicates do not cluster along this axis. PC2 captures the next largest source of variance, which corresponds to the cell-cycle stage-specific transcriptional changes in our synchronized cdc10-129 mutants. Because this variance is directly tied to our experimental manipulation, replicates cluster along PC2. The fact that replicates cluster in PC2 but not PC1 therefore reflects that our synchronization signal is the second largest source of variance in the dataset, and that PC1 is dominated by biological heterogeneity in the unsynchronized controls.

For the differential expression of gene analysis, we calculated the statistics and threshold for the number of differential genes between different groups (Fig. 1b). We also included a comparison of the combined datasets of all the wild-type samples treated at 25 °C, 36 °C and shifted 36 °C→25 °C with all the treated similarly *cdc10-129* samples to identify differential gene expression in the 2 strains regardless of growth conditions. We observe that out of the 1,456 differentially expressed genes, 712 are upregulated in G1 phase, while 744 are upregulated in the G2 phase.

**Fig. 2.**
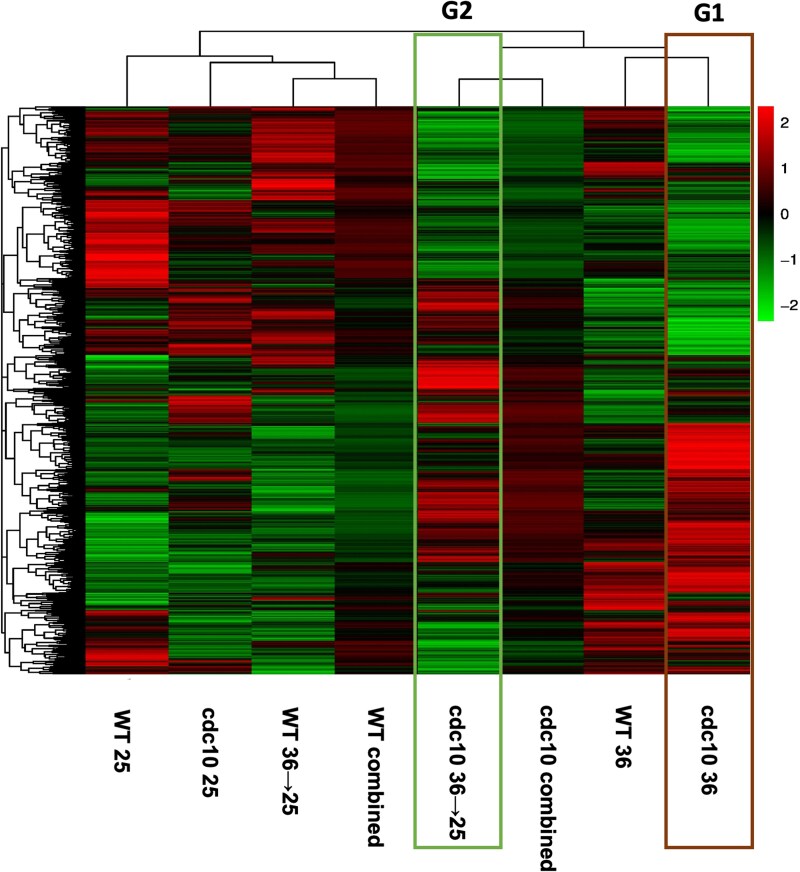
Heat-map representation of differential gene clustering. The overall results of FPKM cluster analysis using the log2(FPKM + 1) value. Red indicates genes with high expression levels, and green indicates genes with low expression levels. The boxes highlight a heatmap representing differential gene expression in G1 and G2 phases of the *cdc10-129* mutant cells.

The differential gene set was generated by combining all the genes in the comparison group that showed differential expression. Cluster analysis was performed on distinct gene sets for more than 2 groups, whereas genes with comparable expression patterns were grouped. We used mainstream hierarchical clustering to cluster the FPKM values of genes and homogenized the row (Z-score). Genes from 18 different samples, pooled into 6 independent groups, were used to generate the heatmap (Fig. 2). Genes that have a similar pattern of expression were grouped on the heatmap. Each grid's color represents the value derived after homogenizing the expression data, typically between −2 and 2. Our clustering data heatmap showed inter-group clustering and inter-sample clustering (Fig. 2 and Supplementary Fig. 7). We observed a clear distinction in the gene expression clustering profile for the cells in G1 phase vs G2 phase. The clear demarcation of the clustering profile in these cells indicates that different cell-cycle stages exhibit differential gene expression.

In our experimental design, to synchronize the cells in G1, we need to treat them at 36 °C for 4 h, and for G2, the cells are shifted back to 25 °C for 150 min. Thus, the differential gene expression observed in the synchronized samples is due to a combination of the cell-cycle stage and temperature shifts. Thus, we have eliminated temperature-related changes observed in wild-type cells treated in the same manner as the cdc10-129 mutants and only consider genes that show variable gene expression in a cell-cycle-dependent manner. We generated co-expression Venn diagrams of different *cdc10-129* samples after accounting for overlapping gene expression in wild-type cells treated under similar conditions. The co-expression Venn diagram shows that the overlapping region between different samples contains a total of 212 genes (Supplementary Fig. 6b). The uniquely identified genes for the 3 different *cdc10-129* samples, namely asynchronous *cdc10-129* at 25 °C, G1 phase-arrested at 36 °C, and G2 phase post-release at 25 °C, are reported as 159, 390, and 721 genes, respectively. 498 genes are co-expressed between the G1 phase cells and the G2 phase cells.

For different experimental conditions, we selected appropriate software for differential gene expression analysis (Supplementary Table 5). We screened genes using a threshold of log2(Foldchange) ≥ 1 and adjusted *P*-value, padj ≤ 0.05. For our analysis, we were only interested in the expression of genes that were differentially expressed between the G1 phase and the G2 phase of the cell cycle. Therefore, we filtered the enrichment results from the raw differential analysis table to identify only the genes of interest (Supplementary Table 10). The goal of this investigation was to identify gene expression patterns that drive the transition from monopolar to bipolar growth in a cell-cycle-dependent manner. Hence, in our analysis, we selected genes that have been shown to play a role in cell polarity. To substantiate our findings, when the number of differential genes discovered was too large, we screened using a stringent threshold criterion and the adjusted *P*-value (FDR) of ≤0.05.

We identified several genes differentially expressed between the G1 and G2 phases of the cell cycle. We also provide a list of top hits for gene expression changes in response to the temperature shift (WT 36 vs WT25→36). For the cell-cycle responsive differentially expressed genes, we only considered the genes unique to the synchronized *cdc10-129* samples, but not to wild-type cells treated under similar conditions. These genes, along with their log2fold change and description, are reported in Table 1. Genes such as *rgs1*, *spk1*, *hsp90*, and *ste11* showed upregulation in the G1 phase. In the G2 phase cells, genes such as *gas1*, *gas5*, *lat1*, *pda1*, *pkd2*, *hob3*, and *cdc15* were upregulated. Our data show that the majority of genes upregulated in the G1 phase are involved in regulating the MAPK pheromone response pathway, protein processing in the endoplasmic reticulum, response to pheromone, and conjugation. Genes upregulated in the G2 phase cells are involved in cell wall organization, plasma membrane maintenance, and glucose metabolism.

**Table 1.** Differential expression of genes in response to the cell cycle and temperature shifts based on log2fold change, *P*-value and padj < 0.05.

Rank	Gene name	Gene ID	Description	log2 Fold change	*P*-value	FDR (padj)
**Cell cycle responsive genes**						
**Genes upregulated in G1 (cdc10 36)**						
1	rgs1	SPAC22F3.12c	Regulator of G-protein signaling Rgs1	+3.06	1.78E−57	4.84E−54
2	mei2	SPAC27D7.03c	RNA-binding protein involved in meiosis Mei2	+2.93	7.82E−40	3.05E−37
3	zta1	SPCC1442.16c	NADPH quinone oxidoreductase/ARE-binding protein (predicted)	+1.33	1.80E−30	4.68E−28
4	SPNCRNA.993	SPNCRNA.993	Intergenic RNA (predicted)	+3.67	3.23E−25	7.05E−23
5	SPBC119.03	SPBC119.03	Human COMT catechol O-methyltransferase homolog 1	+1.49	1.32E−24	2.58E−22
6	scn3	SPAC688.13	TatD DNase family Scn1	+1.67	1.35E−21	2.16E−19
7	SPBC21B10.08c	SPBC21B10.08c	Antibiotic biosynthesis monooxygenase-like domain (predicted)	+1.00	6.73E−19	9.18E−17
8	mat2-Pc	SPMTR.01	Silenced P-specific polypeptide Pc	+1.55	4.53E−16	4.84E−14
9	mas5	SPBC1734.11	DNAJ domain protein Mas5 (predicted)	+1.54	1.08E−14	9.51E−13
10	pss1	SPAC110.04c	Heat shock protein Pss1	+1.39	3.99E−14	3.30E−12
11	cph1	SPAC16C9.05	Clr6 histone deacetylase associated PHD protein-1 Cph1	+1.39	9.58E−14	7.80E−12
12	SPAC2C4.04c	SPAC2C4.04c	Conserved eukaryotic protein	+1.41	5.24E−13	4.08E−11
13	spk1	SPAC31G5.09c	MAP kinase Spk1	+2.46	9.42E−12	6.27E−10
14	bgl2	SPAC26H5.08c	Glucan beta-glucosidase Bgl2 (predicted)	+1.02	1.33E−11	8.54E−10
15	nab3	SPAC3H8.09c	Poly(A) binding protein Nab3 (predicted)	+1.24	1.16E−10	6.54E−09
16	SPBC4F6.17c	SPBC4F6.17c	Mitochondrial heatshock protein Hsp78 (predicted)	+1.52	6.07E−09	2.73E−07
17	cns1	SPAC17A2.04c	HSP chaperone complex subunit Cns1 (predicted)	+1.01	6.32E−09	2.82E−07
18	swc2	SPBP35G2.13c	Swr1 complex subunit Swc2	+1.00	8.45E−09	3.63E−07
19	car2	SPBC21C3.08c	Ornithine transaminase Car2	+1.03	2.11E−08	8.39E−07
20	ppk33	SPCC162.10	Serine/threonine protein kinase Ppk33 (predicted)	+1.81	3.50E−08	1.36E−06
**Genes upregulated in G2 (cdc10 36→25)**						
1	spp27	SPCC285.17	RNA pol I upstream activation factor complex subunit Spp27	−1.57	2.39E−45	1.63E−42
2	SPBC460.01c	SPBC460.01c	Amino-acid permease (unknown)	−1.37	1.70E−31	4.63E−29
3	gas1	SPAC19B12.02c	Cell wall protein Gas1, 1,3-beta-glucanosyltransferase (predicted)	−1.24	3.64E−25	7.63E−23
4	vht1	SPAC1B3.16c	Vitamin H transmembrane transporter Vht1	−1.11	1.07E−24	2.15E−22
5	cdc15	SPAC20G8.05c	Extended Fer/CIP4 (EFC) domain protein Cdc15	−1.75	7.81E−24	1.37E−21
6	SPBC460.05	SPBC460.05	Membrane transporter (predicted)	−1.96	2.86E−20	4.34E−18
7	ecm33	SPAC1705.03c	Cell wall protein Ecm33	−1.11	5.81E−20	8.33E−18
8	plo1	SPAC23C11.16	Polo kinase Plo1	−1.08	2.79E−19	3.91E−17
9	mik1	SPBC660.14	Mitotic inhibitor kinase Mik1	−1.46	1.80E−17	2.28E−15
10	SPBC83.16c	SPBC83.16c	Conserved eukaryotic protein	−1.10	2.66E−17	3.30E−15
11	caf5	SPBC609.04	Spermine family transmembrane transporter Caf5	−1.31	4.14E−17	4.90E−15
12	SPBC460.02c	SPBC460.02c	Eukaryotic translation elongation factor/glutathione S-transferase (predicted)	−1.00	4.44E−17	5.15E−15
13	osr1	SPCC663.06c	Short chain dehydrogenase (predicted)	−1.79	6.00E−17	6.68E−15
14	SPCC1739.01	SPCC1739.01	zf-CCCH type zinc finger protein	−1.00	9.75E−17	1.06E−14
15	mac1	SPAC13G7.04c	Membrane anchored protein Mac1	−1.19	8.19E−16	8.27E−14
16	SPCC306.11	SPCC306.11	Schizosaccharomyces specific protein	−1.85	1.55E−15	1.51E−13
17	ssb1	SPBC660.13c	DNA replication factor A subunit Ssb1	−1.11	1.54E−15	1.51E−13
18	ght5	SPCC1235.14	Hexose transmembrane transporter Ght5	−1.50	4.14E−15	3.89E−13
19	eis1	SPCC63.14	Eisosome assembly protein Eis1	−1.75	5.72E−15	5.28E−13
20	gdh1	SPCC622.12c	NADP-specific glutamate dehydrogenase Gdh1 (predicted)	−1.67	1.07E−14	9.51E−13
**Temperature responsive genes**						
**Genes upregulated at 36 °C (WT 36)**						
1	sti1	SPCC645.14c	Chaperone activator Sti1 (predicted)	+3.09	1.01E−70	5.29E−67
2	SPAC27D7.09c	SPAC27D7.09c	But2 family protein	+3.41	5.79E−70	1.52E−66
3	hsp90	SPAC926.04c	Hsp90 chaperone	+2.76	1.39E−42	2.44E−39
4	ssa2	SPCC1739.13	Heat shock protein Ssa2	+2.90	5.93E−38	6.21E−35
5	SPCC663.09c	SPCC663.09c	Short chain dehydrogenase (predicted)	+1.45	1.62E−28	1.22E−25
6	pgk1	SPBC14F5.04c	Phosphoglycerate kinase Pgk1 (predicted)	+1.69	3.21E−28	2.10E−25
7	aha1	SPBC1711.08	Chaperone activator Aha1 (predicted)	+2.32	8.93E−27	5.20E−24
8	SPCC338.06c	SPCC338.06c	Heat shock protein Hsp20 family (predicted)	+1.57	8.43E−24	4.42E−21
9	ssc1	SPAC664.11	Mitochondrial heat shock protein Hsp70	+1.46	4.88E−16	1.97E−13
10	wos2	SPAC9E9.13	p23 homolog, predicted co-chaperone Wos2	+2.26	7.30E−15	2.55E−12
11	cnx1	SPAC3C7.11c	Calnexin Cnx1	+1.32	1.39E−14	4.55E−12
12	fes1	SPBC3B9.01	Hsp70 nucleotide exchange factor Fes1 (predicted)	+2.01	2.80E−13	7.33E−11
13	hsp3104	SPAC11D3.13	ThiJ domain protein	+1.26	3.77E−13	9.24E−11
14	ats1	SPAC1002.07c	N-acetyltransferase Ats1 (predicted)	+1.32	3.88E−13	9.24E−11
15	SPNCRNA.855	SPNCRNA.855	Intergenic RNA (predicted)	+4.45	9.71E−12	1.77E−09
16	hsp16	SPBC3E7.02c	Heat shock protein Hsp16	+2.64	9.81E−12	1.77E−09
17	SPAC23D3.12	SPAC23D3.12	Inorganic phosphate transmembrane transporter (predicted)	+1.02	1.32E−11	2.30E−09
18	grx1	SPAC4F10.20	Glutaredoxin Grx1	+1.59	3.47E−11	5.35E−09
19	SPBC30D10.14	SPBC30D10.14	Dienelactone hydrolase family (predicted)	+1.86	9.93E−11	1.44E−08
20	mug35	SPAC22H12.01c	Schizosaccharomyces specific protein Mug35	+2.10	1.85E−10	2.55E−08
**Genes upregulated after shift to 25 °C (WT 36→25)**						
1	SPAC869.02c	SPAC869.02c	Nitric oxide dioxygenase (predicted)	−1.69	2.45E−38	3.21E−35
2	mae2	SPCC794.12c	Malic enzyme/malate dehydrogenase, Mae2	−1.80	1.30E−29	1.14E−26
3	SPCC569.05c	SPCC569.05c	Spermidine family transmembrane transporter (predicted)	−1.89	4.49E−19	2.14E−16
4	hta2	SPAC19G12.06c	Histone H2A beta	−1.22	1.41E−16	6.18E−14
5	gsf1	SPBC15D4.02	Transcription factor, zf-fungal binuclear cluster type Gsf1	−1.37	7.81E−16	2.92E−13
6	hht3	SPBC1105.11c	Histone H3 h3.3	−1.03	8.72E−14	2.69E−11
7	SPAC5H10.03	SPAC5H10.03	Phosphoglycerate mutase family	−1.11	1.03E−13	3.01E−11
8	ssn6	SPBC23E6.09	Transcriptional corepressor Ssn6	−1.26	2.03E−12	4.43E−10
9	SPBC660.05	SPBC660.05	WW domain containing conserved fungal protein	−2.81	4.27E−12	8.30E−10
10	SPAC1039.02	SPAC1039.02	Phosphoprotein phosphatase (predicted)	−1.41	1.73E−11	2.93E−09
11	SPBC1E8.05	SPBC1E8.05	Conserved fungal protein	−1.10	2.48E−11	4.05E−09
12	gly1	SPAC23H3.09c	Threonine aldolase Gly1 (predicted)	−1.03	3.24E−11	5.14E−09
13	gar2	SPAC140.02	Nucleolar protein required for rRNA processing	−1.23	4.70E−11	7.04E−09
14	isp3	SPAC1F8.05	*Schizosaccharomyces pombe* specific protein Isp3	−1.31	3.96E−10	5.32E−08
15	gln1	SPAC23H4.06	Glutamate-ammonia ligase Gln1	−1.48	4.24E−10	5.56E−08
16	tos4	SPAP14E8.02	FHA domain protein Tos4 (predicted)	−1.16	1.05E−09	1.31E−07
17	amt1	SPCPB1C11.01	Ammonium transmembrane transporter Amt1	−1.40	1.52E−09	1.77E−07
18	cdc18	SPBC14C8.07c	MCM loader	−1.18	2.12E−09	2.41E−07
19	SPAC2E1P3.05c	SPAC2E1P3.05c	Fungal cellulose binding domain protein	−1.33	2.28E−09	2.50E−07
20	isp6	SPAC4A8.04	Vacuolar serine protease Isp6	−1.07	2.29E−09	2.50E−07

The values for the log2fold change are indicated for all the genes. “+” indicates upregulated and “−” indicates downregulated genes.

### GO and KEGG enrichment analysis of differentially expressed genes identifies pathways for polarity control

**Fig. 3.**
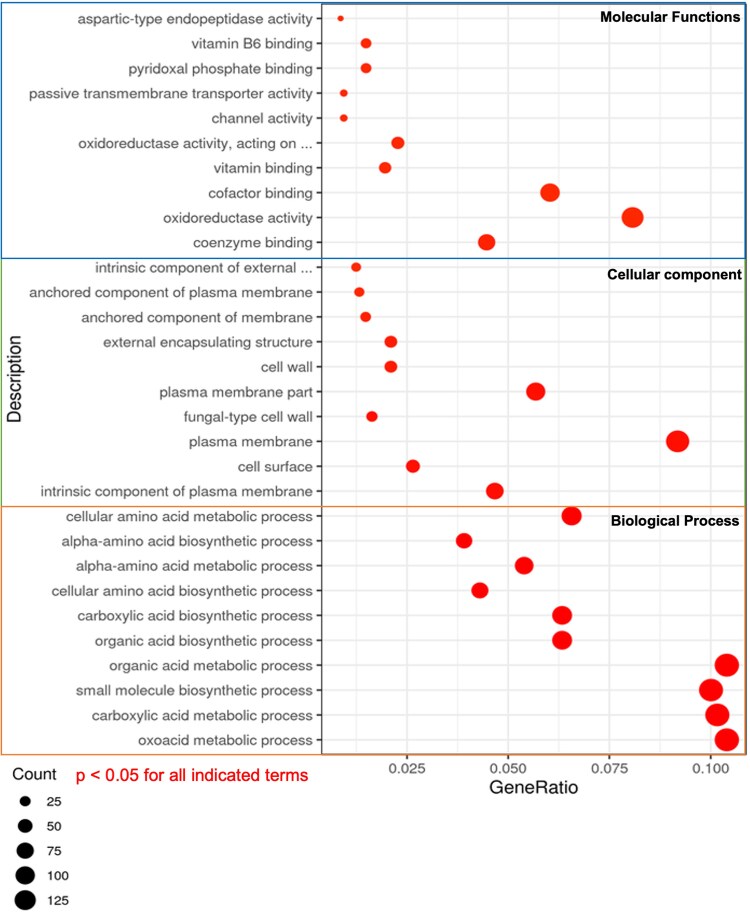
GO enrichment analysis: GO enrichment analysis scatter plot with the 30 most significant GO terms in G1 vs G2 population. The abscissa is the ratio of the number of differential genes linked with the GO term to the total number of differential genes, independent of directionality of the expression change, and the ordinate is the GO term. The size of the point represents the number of genes annotated to a specific GO term. The 3 different categories of biological process, cellular component, and molecular function are represented with orange, green, and blue outlines.

Next, we performed GO enrichment analysis (http://www.geneontology.org/) to present gene properties of the cellular components, molecular functions, and biological processes across all species. GO terms with padj-value ≤ 0.05 are considered significantly enriched. In our GO enrichment analysis, the 30 most significant terms were selected for each comparison group. The data for GO terms for genes differentially expressed between cells growing in the G1 phase and G2 phase are shown in Fig. 3. We have also reported in Supplementary Figs. 8 and 9, the GO terms upregulated for G1 and G2 phase cells. We observe that most of the GO terms associated with differentially expressed genes between G1 and G2 phase cells regulate various biosynthetic and metabolic pathways, including cell wall organization and plasma membrane maintenance. The pathways associated with differentially expressed genes in G1 phase cells were involved in regulating protein folding, rRNA processing, cellular response to pheromone, 90s preribosome, unfolded protein binding, and heat-shock protein binding. We observed genes upregulated in the G2 phase that control oxalic acid metabolism, organic acid metabolism, fungal cell wall organization, organelle and endoplasmic reticulum subcompartments, cofactor and coenzyme binding.

**Fig. 4.**
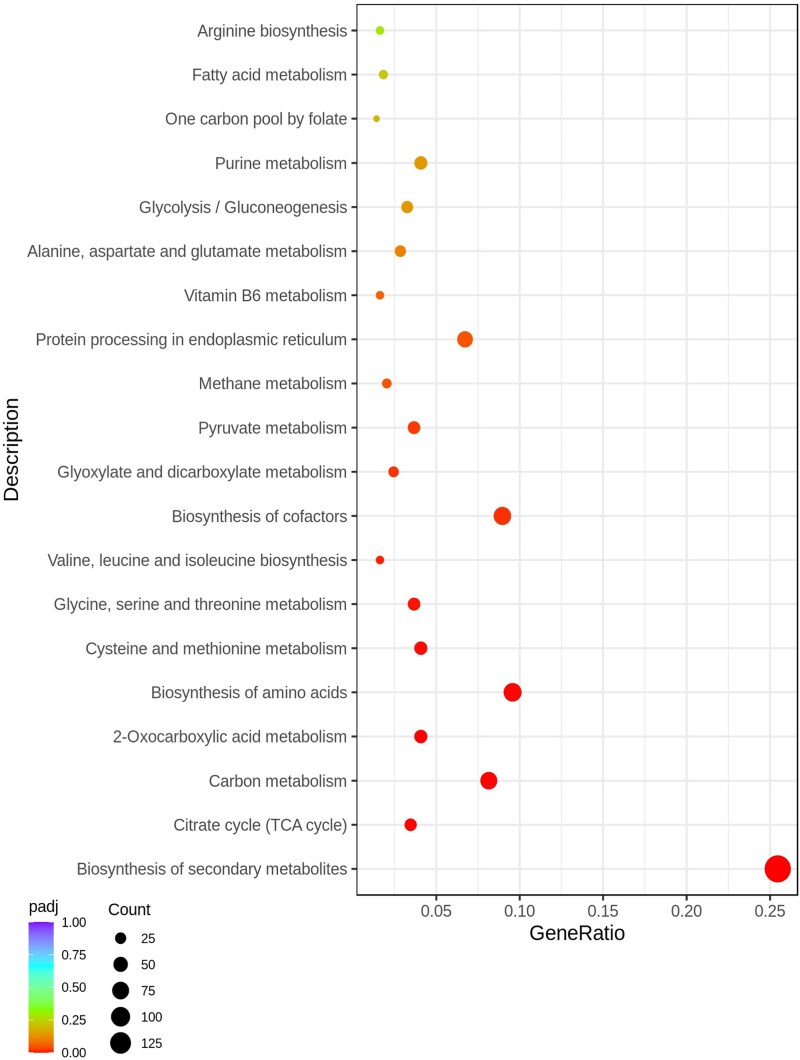
KEGG pathway analysis: KEGG enrichment scatter plot with 20 most significant pathways showing differential expression in G1 and G2 phase cells. The abscissa in the graph is the ratio of the number of differential genes on the KEGG pathway to the total number of differential genes, and the ordinate is the KEGG pathway. The size of the point represents the number of genes annotated to a specific GO term, and the color from red to purple represents the significance level of the enrichment.

The KEGG enrichment analysis for cells growing in G1 and G2 phases of the cell cycle displays a scatter plot of up to 20 of the most significant pathways, differentially regulated in G1 phase cells and G2 phase cells (Fig. 4). Twenty upregulated pathways for genes associated with G1 phase and G2 phase of the cell cycle are reported in Supplementary Figs. 10 and 11 and Supplementary Tables 11 and 12. Some of the important pathways with upregulated genes in the G1 phase include protein processing in the endoplasmic reticulum, biosynthesis of cofactors, vitamin B6 metabolism, and RNA degradation. Upregulated genes associated with the G2 phase of the cell cycle include the TCA cycle and various metabolic pathways. These data suggest that cells growing in the G2 phase regulate the metabolism of glucose and various amino acids to prepare the cell to undergo bipolar growth in the presence of available nutrients.

GSEA (Subramanian et al. 2005) analysis was performed for all 16 comparison groups. We have reported upregulated genes in G1 phase and G2 phase cells in Supplementary Table 13. Some of the genes that are differentially regulated in these cell-cycle stages include *hsp90*, *spk1*, *rgs1*, *cdc15*, and *gas1*. Most of these genes are involved in regulating the pheromone response pathway, metabolic pathway, and pathways controlling the cell wall organization and plasma membrane maintenance. The data obtained via GSEA ranking agrees with our enrichment analysis in GO and KEGG pathway analysis.

### PPI analysis of differentially expressed genes suggests clustering of polarity pathways

**Fig. 5.**
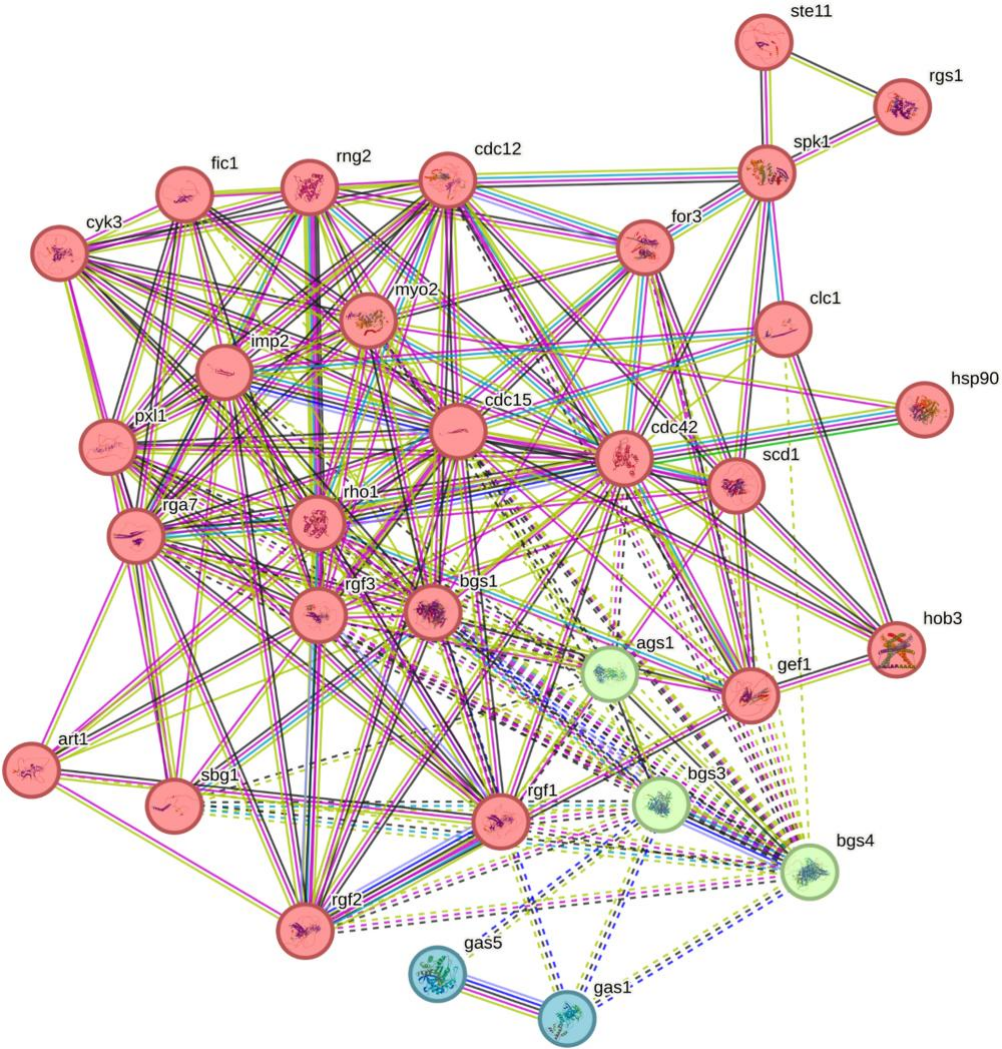
PPI analysis for upregulated and downregulated genes in the G1 phase cells. PPI network generated using STRING database showing genes up- and downregulated in G1 phase. Input genes were mapped to the STRING database. Additional interacting proteins not present in the original input list were incorporated by STRING to extend the network. A total of 31 nodes, including 189 edges, were identified representing both the downregulated and upregulated genes. The average node degree was estimated to be 12.2, whereas the average local clustering coefficient is 0.755. PPI enrichment value was estimated to be < 1.0e−16. Three basic clusters appear in the PPI analysis using *k*-means clustering. Red outline around the gene indicates genes involved in cell polarization, cytokinetic process, cell-cortex region, contractile ring formation, and small GTPase-mediated signal transduction. Green outline around the gene indicates genes involved in fungal-type cell-wall polysaccharide synthesis, glycosyl transferase, primary cell septum biogenesis, and UDP glycosyl transferase activity. Blue outline around the gene indicates genes involved in fungal-type cell wall (1->3)-beta-D-glucan biosynthesis. Edges represent PPI, with solid lines indicating interactions supported by “direct” or high-confidence evidence while dashed lines indicate predicted or indirect interactions.

**Fig. 6.**
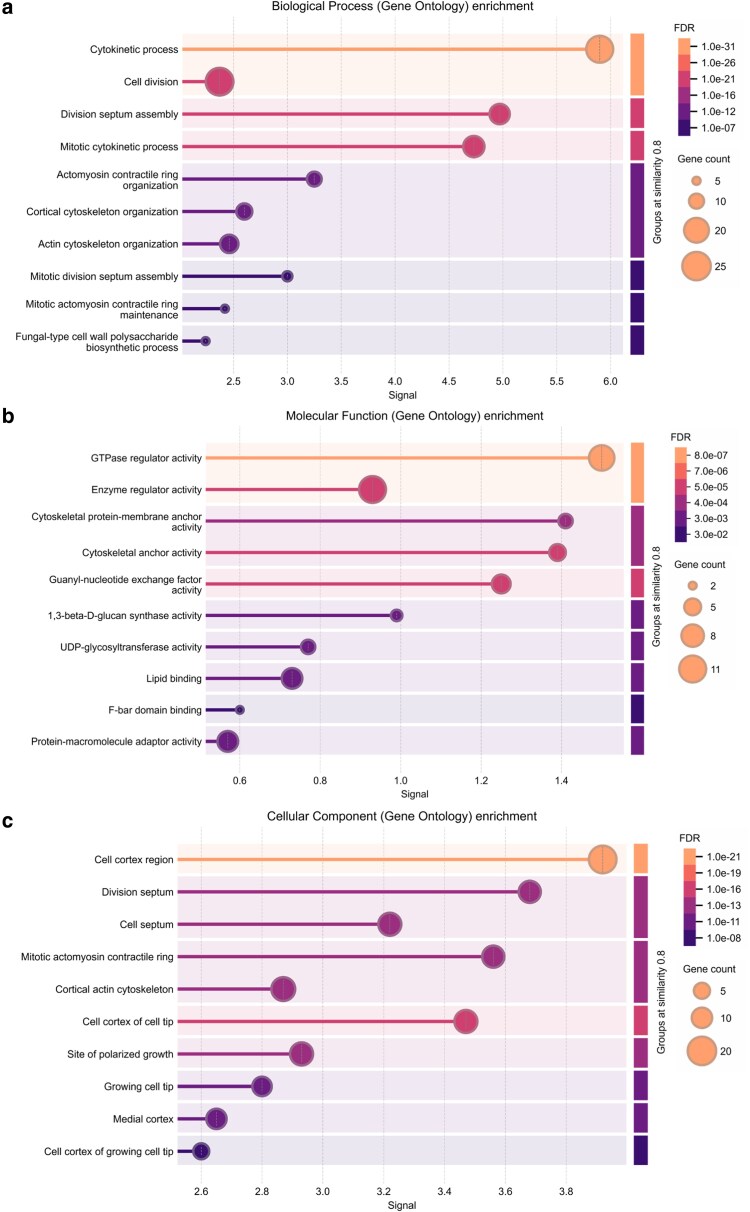
PPI analysis using string for downregulated and upregulated genes in the G1 phase *cdc10-129* cells. Top 10 GO enrichment pathways identified by PPI analysis in the following categories: a) Biological process, b) molecular functions, c) cellular component.

Protein interaction patterns between differentially expressed genes were identified through the analysis of the STRING protein interaction database (Szklarczyk et al. 2019). Using the STRING database, we constructed networks that visualize how proteins from differentially expressed genes interact with each other. As observed in Figs. 5 and 6, we used upregulated and downregulated genes in G1 and G2 phases of the cell cycle to provide a network with molecular species represented as nodes and intermolecular interactions represented as edges. Our data for PPI analysis show that the genes upregulated in G1 and G2 phases have interacting partners in either upstream or downstream pathways controlling cell polarization. These genes likely work in concert to globally regulate cell polarization in a cell-cycle-dependent manner. Upon further analysis of PPI for G1 and G2 phase cells, we identified the biological processes, cellular components, and molecular functions similar to those observed in our GO analysis (Supplementary Figs. 12–15). We identified upregulation of the *spk1-byr2-ste11* hub in G1 phase cells (Supplementary Fig. 12). These genes are involved in pheromone response and G1-arrest due to nutritional starvation (Nielsen and Davey 1995; Halova et al. 2021). The biological processes, molecular functions and cellular components upregulated in G1 are listed in Supplementary Fig. 13. In the G2 phase cells, we identified the *cdc15*-*hob3*-*rho1*-*bgs1* hub that is involved in regulating pathways responsible for cell wall organization Supplementary Fig. 14. The biological processes, molecular functions, and cellular components upregulated in G2 identified pathways in positive regulation of cellular component biogenesis, plasma membrane maintenance, fatty acid metabolism, and TCA cycle regulation (Supplementary Fig. 15) (Arellano et al. 1999b; Coll et al. 2007; Cortes et al. 2002, 2007; Hercyk and Das 2019). These findings indicate upregulation of pathways that promote cell growth and expansion in G2.

### Validating cell-cycle-dependent gene expression profiles of polarity pathways

To obtain cell-cycle-specific mRNA, we used the *cdc10-129* temperature-sensitive mutant. In our experimental design, the mutant at high temperature (36 °C) is arrested in G1 and released to G2 when grown under permissive conditions (25 °C). It is possible that the difference in the temperature conditions impacted gene expression in the *cdc10-129* mutants. However, KEGG analysis of our data in wild-type cells grown at 25 °C compared to 36 °C displayed very different pathways compared to our data from the G1 and G2 phase cells (Supplementary Fig. 16). We identified close to 95 statistically significant temperature-responsive transcripts (*P* < 0.05). GO enrichment analysis revealed significant changes in genes associated with ribosomal functionality and SNARE-mediated vesicular transport mechanisms. Notably, no statistically significant alterations were observed in transcripts governing MAPK signaling cascades, cell-cycle regulatory networks, or cell polarity determinants in wild-type specimens.

**Fig. 7.**
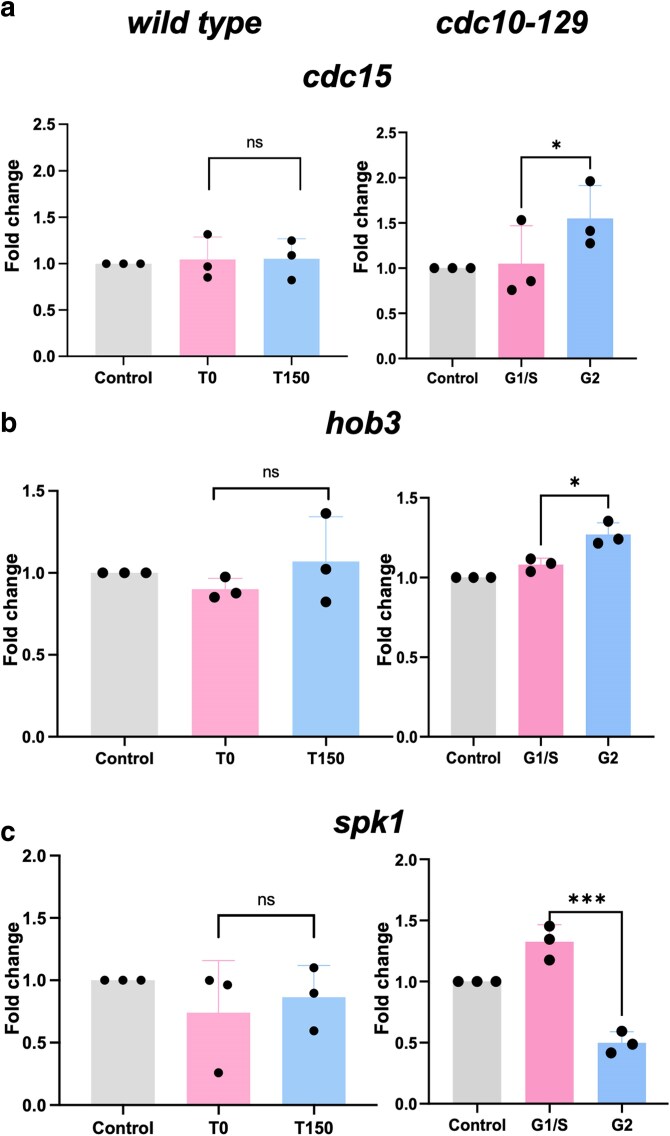
qPCR expression profile for *cdc15*, *hob3*, and *spk1* in wild-type *and cdc10-129* mutant cells. Fold change in mRNA expression in wild-type *and cdc10-129* cells in different cell-cycle stages for *cdc15* (a), *hob3* (b), and *spk1* (c). Grey bar represents control asynchronous cells in *wild-type* and *cdc10-129* mutant cells at 25 °C. The pink bar represents G1 phase cells in *cdc10-129* mutant cells and asynchronized wild-type cells growing at 36 °C. The light blue bar represents G2 phase cells in *cdc10-129* mutant cells, as well as asynchronous wild-type cells at 2 and 3 h after shift to 25 °C. The error bar represents the standard deviation, *n* = 3 replicate experiments. Statistical analysis between the 2 conditions was performed by paired Student's *t*-test, n.s. is not statistically significant, *P < 0.05, ***P < 0.0007.

To further validate our gene expression data, we performed qPCR for *spk1*, *cdc15*, and *hob3* with reference to the β-actin gene *act1* in wild-type cells and synchronized *cdc10-129* cells (G1 at 36 °C and G2 at 25 °C) (Tormos-Perez et al. 2016). The *act1* gene demonstrates remarkable transcriptional stability across diverse physiological conditions and hence was selected as the qPCR reference gene. Our high-throughput RNA sequencing analysis revealed minimal expression variability (coefficient of variation <0.1) across experimental conditions, regardless of temperature and cell-cycle phase. Statistical analysis of normalized read counts demonstrated no significant differential expression (*P* > 0.05, FDR-adjusted) of *act1* under any tested condition. This robust transcriptional stability, coupled with its abundant expression levels (FPKM >500), establishes *act1* as an optimal internal control for relative quantification in the qPCR analyses. Using qPCR, we show that in the wild-type cells, irrespective of the temperature, *cdc15*, *hob3*, and *spk1* levels do not change. In contrast, *cdc10-129* mutants in G2 phase show upregulation of *cdc15* and *hob3* (Fig. 7a and b). Similarly, during the G1 phase in the *cdc10-129* mutant, the *spk1* gene was upregulated (Fig. 7c). Together, these results further indicate that the differential gene expression data in G1 and G2 phase cells is not due to the effects of the different temperatures.

### Cell-cycle-dependent gene expression patterns correspond to cell polarization patterns

**Fig. 8.**
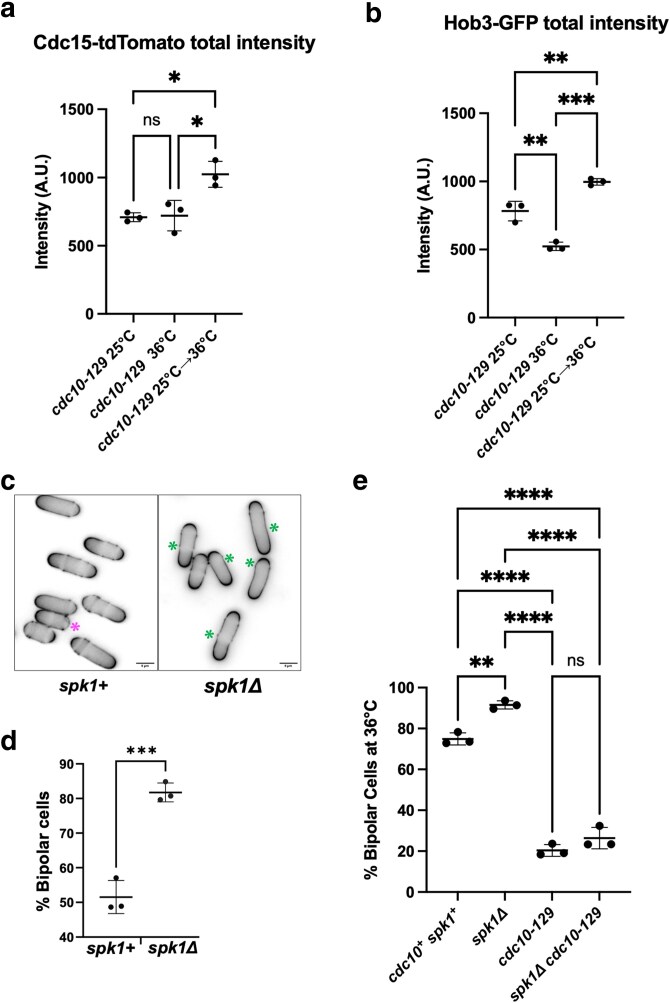
Protein levels and polarity index analysis validate gene expression data. Total mean intensity of Cdc15-tdTomato a) and Hob3-GFP b) in *cdc10-129* mutants under asynchronous, G1 arrested and G2 released conditions. *N* = 3, *n* > 50. Statistical analysis 2-way ANOVA with multiple comparisons. *P < 0.05, **P < 0.01, ***P < 0.001. c) *spk1+* and *spk1Δ* cells at 25°C. d) Quantitative analysis of percentage bipolar cells in *spk1+* cells and *spk1Δ* at 25 °C. *N* = 3, *n* > 100. Statistical analysis, unpaired *t*-test. ***P≤ 0.0007. Scale bar, 5μm. e) Quantitative analysis of percentage bipolar cells in *cdc10+ spk1+*, *spk1Δ*, *cdc10-129*, and *cdc10-129 spk1Δ* cells arrested at 36 °C for 4 h. *N* = 3, *n* > 100. Statistical analysis 1-way ANOVA with multiple comparisons. **P < 0.01, ****P < 0.0001.

Our bioinformatics data identified 2 Bin-Amphiphysin-Rvs (BAR) domain-containing genes, the F-BAR gene *cdc15* and the N-BAR gene *hob3*. The F-BAR *cdc15* is required for cytokinesis and is also a component of the endocytic complex (Hercyk and Das 2019). Hypomorphic mutants of *cdc15* are monopolar, while hypermorphic mutants are precociously bipolar (Hercyk and Das 2019). Cdc15 promotes bipolarity via the recruitment of the Cdc42 GEF Gef1 to the growing ends (Hercyk and Das 2019). The *hob3* gene is also involved in Gef1-mediated Cdc42 activation (Routhier et al. 2001; Coll et al. 2007). Cells lacking *hob3* mislocalize Gef1 and show decreased Cdc42 activation. Gef1 helps establish Cdc42 positive feedback activation and is required for new-end-take-off leading to bipolar growth (Coll et al. 2007; Hercyk et al. 2019; Hercyk and Das 2019). Thus, the upregulation of *cdc15* and *hob3* in the G2 phase of the cell cycle agrees with their role in promoting bipolar growth. To further validate our data, we analyzed total Cdc15-tdTomato and Hob3-GFP levels in *cdc10-129* mutants arrested in G1 at 36 °C and released to G2 after 150 min at 25 °C (Fig. 8a and b). We find that total mean intensity of Cdc15-tdTomato and Hob3-GFP increase in cells in G2 compared to G1 cells in agreement with the gene expression data.

Our RNA seq data analysis also showed that the MAPK pheromone response pathway is upregulated in the G1 phase. We identified the MAP kinase Spk1 as one of these upregulated genes. The gene *spk1*, a nutritional-stress-related MAP kinase, regulates response to pheromone, conjugation, and mating pathways (Nielsen and Davey 1995; Waszczak et al. 2019; Halova et al. 2021; Sieber et al. 2023). Spk1 kinase also promotes G1 to G0 transition in response to nitrogen starvation (Halova et al. 2021). Thus, it is expected that this pathway, including Spk1, is upregulated in G1 phase cells. However, it is unclear if this pathway is involved in the transition from monopolar to bipolar growth. We asked if upregulation of *spk1* in G1 prevents monopolar to bipolar transition. A previous report investigating all fission yeast kinases involved in the transition from monopolar to bipolar growth identified *spk1*Δ cells as monopolar (Koyano et al. 2010). However, we find that in *spk1Δ* mutant cells at 25 °C, about 80% of the cells were bipolar compared to *spk1+* cells with 51% bipolar cells (Fig. 8c and d). This indicates that in the absence of the *spk1* gene, cells initiate bipolar growth prematurely. This suggests that the high *spk1* levels in G1 phase cells prevent the transition to bipolar growth.

While we find that *spk1Δ* cells are precociously bipolar, we asked if low Spk1 levels alone were sufficient to promote bipolarity. To test this, we analyzed the polarity index of *spk1Δ cdc10-129* arrested at 36 °C compared to *cdc10-129* under similar conditions. We find that in both mutants, cells arrest in G1 with decreased bipolarity index (Fig. 8e). It is possible that in addition to Spk1 levels, other factors upregulated in G2, such as pathways in positive regulation of cellular component biogenesis, plasma membrane maintenance, fatty acid metabolism, and TCA cycle regulation, are required for bipolar growth. One caveat of our experimental condition is that to arrest cells in G1, the cells are exposed to high temperature. This may trigger other stress pathway genes that interfere with bipolar growth. Further analysis of how Spk1 promotes bipolar growth will determine the molecular details of how this relates to the cell-cycle stage.

## Discussion

The initial step in cell polarization follows cues generated post-cell division, leading to spatiotemporal organization of polarity proteins for the regulation of the cytoskeleton and cell growth (Sohrmann and Peter 2003; Li and Gundersen 2008; Orlando and Guo 2009; St Johnston and Ahringer 2010; McCaffrey and Macara 2012; Campanale et al. 2017; Rappel and Edelstein-Keshet 2017; Chiou et al. 2021; Pino et al. 2021). In fission yeast, spatiotemporal regulation of Cdc42 drives polarized growth. Despite the small subset of proteins regulating Cdc42 self-organization at the growth site (Das et al. 2012; Hercyk et al. 2019), the underlying principles of cell-cycle-dependent polarized growth remain unclear.

In the current work, we explore the major pathways controlling the transition from monopolar to bipolar growth in a cell-cycle-dependent manner. The transition to bipolar growth only occurs when the cells enter the G2 phase of the cell cycle (Hagan et al. 2016). To identify the factors that enable this transition, we investigated the gene expression pattern of cell-cycle-arrested G1 and G2 phase cells using the *cdc10-129* mutant. Our gene quantification analysis showed intragroup clustering in synchronized *cdc10-129* cells in G1 and in G2 phase. However, there was significant intergroup variability in expression patterns between G1 phase cells and G2 phase cells. Gene clustering heatmap shows differentially regulated gene expression for G1 and G2 phase cells. We found that between these specific groups, a total of 712 genes were upregulated in G1 phase cells, while 744 genes were upregulated in G2 phase cells. Our analysis for the log2fold expression change showed that genes such as *spk1*, *rgs1*, *hsp90*, *lat1*, *pda1*, *hob3*, *cdc15*, *rho1*, and *pkd2* were differentially expressed between the G1 phase and G2 phase of the cell cycle. Using GO analysis, we categorized the genes into 3 distinct groups: Biological Process (BP), Molecular Functions (MF), and Cellular Components (CC). Our GO enrichment analysis and KEGG pathway analysis showed that genes upregulated in G1 phase were involved in controlling protein folding, 90s preribosomes, and rRNA methyltransferase activity. Genes upregulated in G2 phase cells were involved in controlling various metabolic pathways, the TCA cycle, plasma membrane maintenance, cell wall organization, and transmembrane transporter activity. GSEA analysis was performed to validate whether the prior gene set was significantly different between the 2 biological states. The GSEA data agreed with the GO enrichment and KEGG pathway analysis.

PPI analysis was performed for the genes with significant differential expression between the G1 and G2 phases of the cell cycle. Our data show that genes like *spk1*, *rgs1*, and *hsp90* were upregulated in the G1 phase of the cell, which is upstream of the polarity complex. These genes are known to function in the stress response pathway, where Spk1 is the MAP kinase for nutritional stress (Nielsen and Davey 1995; Yamamoto et al. 2004; Waszczak et al. 2019; Halova et al. 2021; Sieber et al. 2023). Interestingly, we did not observe cell-cycle-dependent differential gene expression of the stress response MAP kinase Sty1, which promotes depolarization under stress (Mutavchiev et al. 2016; Salat-Canela et al. 2021; Doyle et al. 2025). In G2 phase cells, genes such as *hob3* and *cdc15*, which indirectly control Cdc42 activation via Gef1, were upregulated. Hob3 is an N-BAR domain-containing protein that binds and recruits Gef1 to its site of action (Coll et al. 2007; Rincon et al. 2007). Similarly, Cdc15 is an F-BAR domain-containing protein that recruits Gef1 to the site of action. Lack of *hob3* or hypomorphic *cdc15* results in monopolar cells due to the inability of Gef1-mediated Cdc42 activation in these mutants (Coll et al. 2007; Hercyk and Das 2019). Thus, upregulation of *cdc15* and *hob3* in G2 phase cells agrees with the known roles of these genes in promoting bipolar growth. In a previous report analyzing cell-cycle-dependent gene expression analysis, Hob3 was identified to be upregulated in G2, similar to our findings (Peng et al. 2005). In the same analysis, as well as others, Cdc15 levels were shown to be highest in M phase cells while gradually decreasing in G1 and subsequently appear to increase in late G2 (Fankhauser et al. 1995; Zilahi et al. 2000; Rustici et al. 2004; Peng et al. 2005). Under normal conditions in fission yeast, the G1 phase is fairly short. Given our experimental condition where the cells arrest in G1 for 4 h, we are able to show how Cdc15 levels change under prolonged G1.

The cell-cycle-dependent gene expression patterns of the nutritional stress response pathways are in agreement with the fact that the G1 arrest occurs in response to nitrogen starvation (Hagan et al. 2016; Halova et al. 2021). Cells growing in the G1 phase can have 2 different fates depending upon the environment they are in: either they enter the S phase and eventually the G2 phase in a stress-free environment, or they enter the quiescent G0 phase under nutritional starvation. Based on our findings, we hypothesize that the nutritional stress pathways are upregulated by default in the G1 phase of the cell cycle, where the cells are monopolar. In the absence of starvation, stress pathways are downregulated, and the cells enter G2, resulting in bipolar growth. Indeed, we find that in cells lacking *spk1*, the transition to bipolar growth occurs prematurely, indicating that *spk1* expression promotes monopolar growth. High Spk1 levels in G1 cells were also identified in the Peng et al. study of cell-cycle-dependent gene expression (Peng et al. 2005).

Cell-cycle-dependent regulation of cell polarity is likely mediated by multiple regulatory modules. While our data identify how gene expression patterns relate to the transition from monopolar to bipolar growth, we do not rule out the possibility of the role of cell-cycle-dependent kinases in this process. However, it should be noted that even during monopolar growth in wild-type cells, the Cdc42 dynamics at the growing ends are not significantly different from those in bipolar cells (Das et al. 2012). The major regulators of Cdc42, such as the GEFs Scd1 and Gef1, the scaffold Scd2, and the negative feedback kinase Pak1, all localize to the monopolar end, resulting in Cdc42 activation at that end. Our data suggest that the transition to bipolar growth requires a global change in growth regulation rather than specific molecular changes in the Cdc42 regulators. Indeed, our gene expression data show upregulation of cellular component biogenesis, cell wall organization, and plasma membrane maintenance pathways in the G2 phase. While the Cdc42 pathway signals and activates growth, for the cell to successfully increase in size, it requires sufficient extension of the plasma membrane and cell wall biogenesis. It is possible that upregulation of these pathways in addition to Cdc42 activation is required for the successful transition to bipolar growth. Further investigations will determine if and how stress response pathways change in the plasma membrane profile, and cell wall reorganization regulates polarity.

Our data indicate that the transition from monopolar to bipolar growth is regulated at multiple levels within the cell. It is conceivable that bipolar growth not only requires activation of the Cdc42 pathway at the 2 growing ends, but that the cell generates sufficient material via anabolic pathways for the 2 cell ends to grow effectively. Indeed, we observed upregulation of cell wall biogenesis and membrane remodeling pathways in the G2 phase, where bipolarity occurs. The transition from G1 to G2 requires a nutritional starvation-free environment. Here, we show that the nutritional stress response pathway that is known to prevent G1 phase transition also impacts the transition to bipolar growth. Upregulation of anabolic activities in G2 phase cells promotes bipolar growth. Further investigations will reveal whether the stress response pathways downregulate anabolic activity thus preventing bipolar growth or whether these 2 processes act together in parallel to promote bipolarity. In nature, fission yeast cells typically form pseudohyphae where they grow in a monopolar manner, end-to-end, toward the nutrition source (Amoah-Buahin et al. 2005; Borup 2006). Under laboratory conditions, fission yeast cells growing in rich media display bipolar growth. Our findings indicate how cell polarity is regulated at the systemic level, where growth conditions and cell-cycle stage play a critical role.

## Supplementary Material

jkag126_Supplementary_Data

## Data Availability

The Raw and processed data are available in NCBI GEO with accession number GSE326505 and are publicly available at https://www.ncbi.nlm.nih.gov/geo/query/acc.cgi?acc=GSE326505. All supporting data can be found in the article and supporting information. Supplemental material available at G3 online.

## Software information

The software used in the analysis for sequencing data is available as Supplementary Table 5. The raw data result file decompression and format description are summarized in Supplementary Table 14.
