# Supplementary material for: Differential gene expression drives cell-cycle-dependent transition from monopolar to bipolar growth in Schizosaccharomyces pombe
Source: G3 (Bethesda). 2026 May 11;16(7):jkag126. doi: 10.1093/g3journal/jkag126 (PMC13334187; doi:10.1093/g3journal/jkag126)
Supplement: jkag126_Supplementary_Data [file jkag126_supplementary_data.zip › Supplementary_Figures_G3-2026-406766.pdf]

Supplementary Figure 1

a

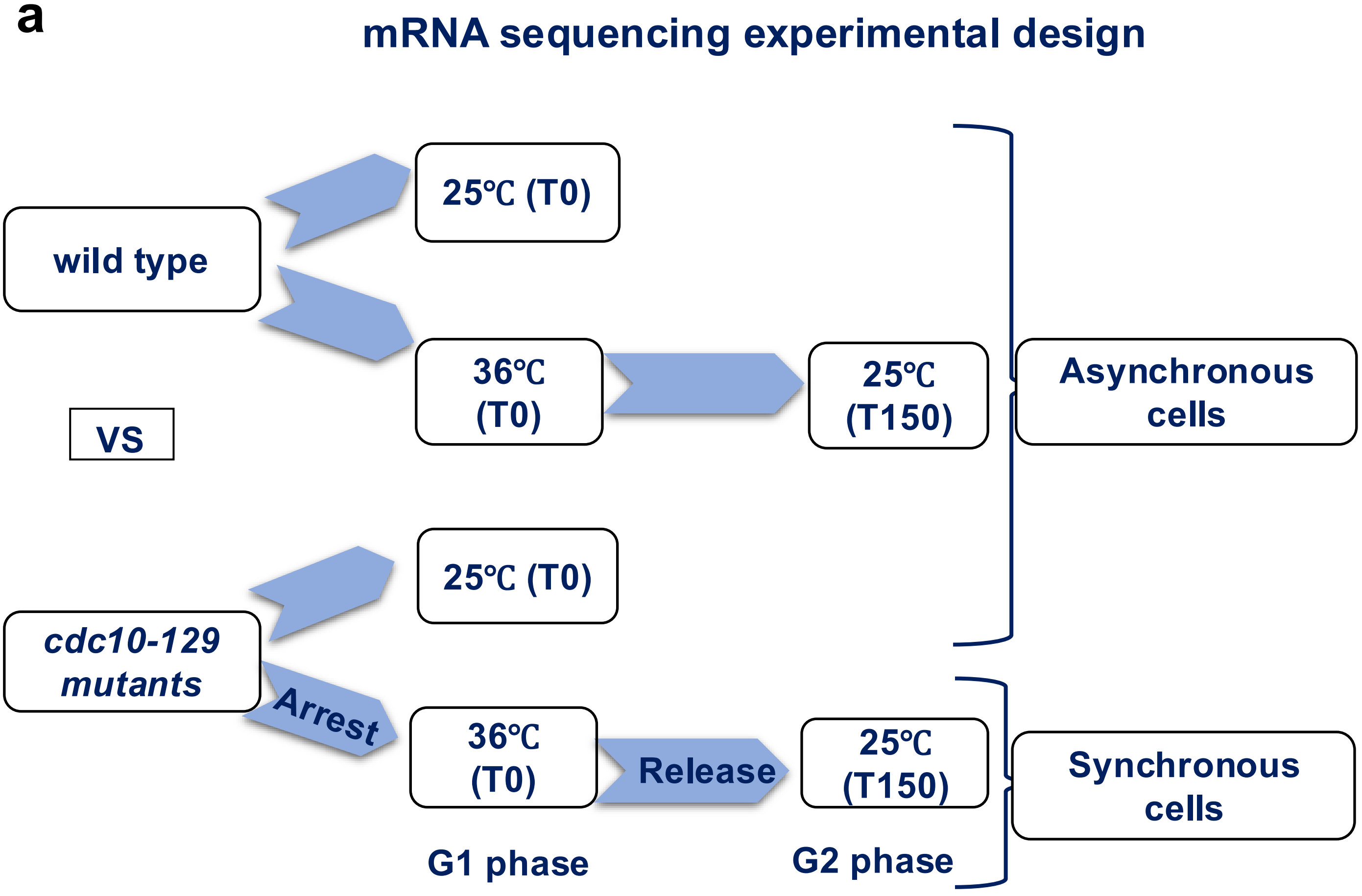

b

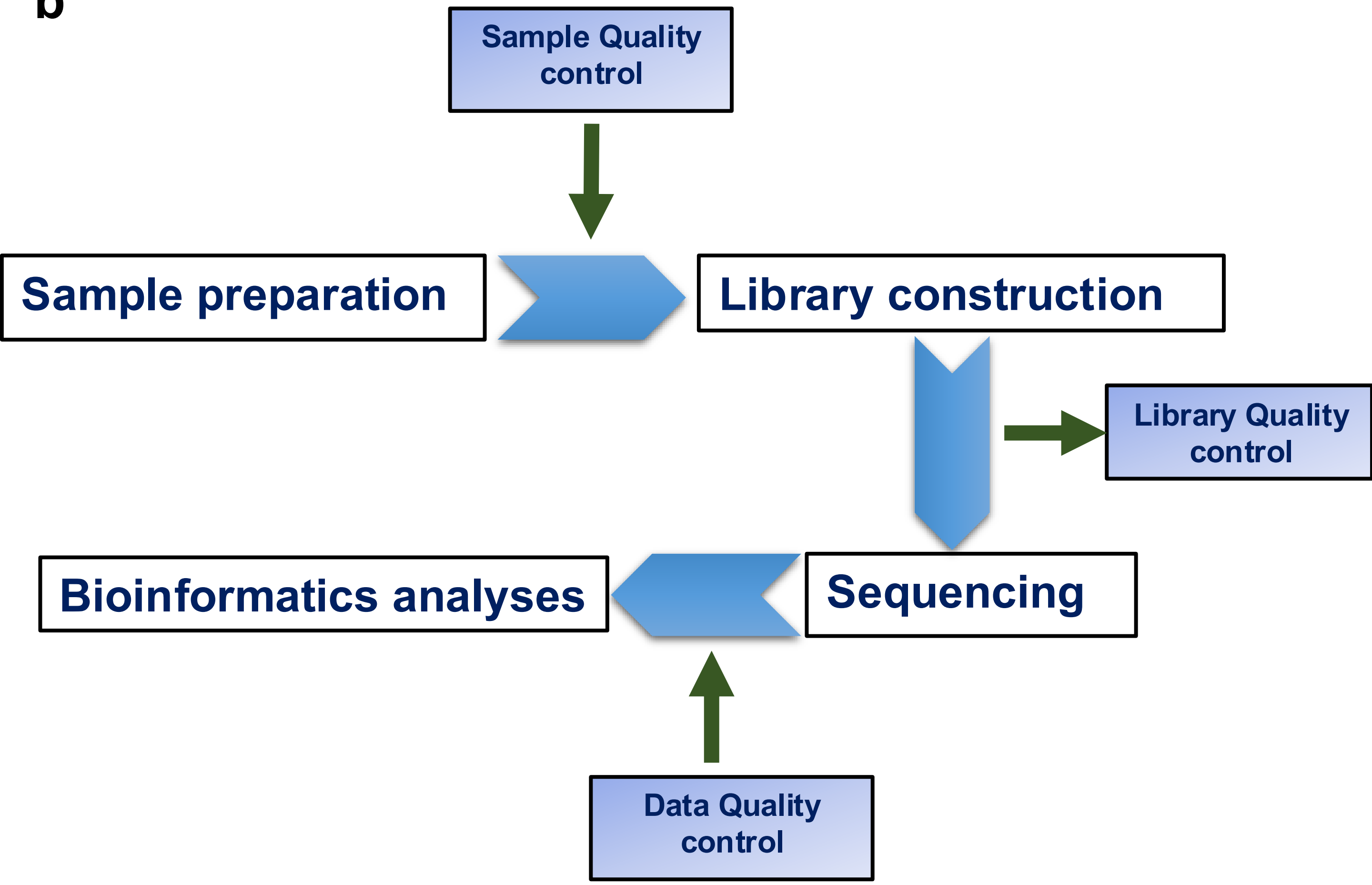

Supplementary Figure 1: Experimental design for high-throughput mRNA sequencing and bioinformatic analysis. a) High-throughput mRNA sequencing using *cdc10-129* synchronized cells. b) Step-wise illustration of RNA sequencing via Illumina platform based on the mechanism of sequencing by synthesis.

Supplementary Figure 2

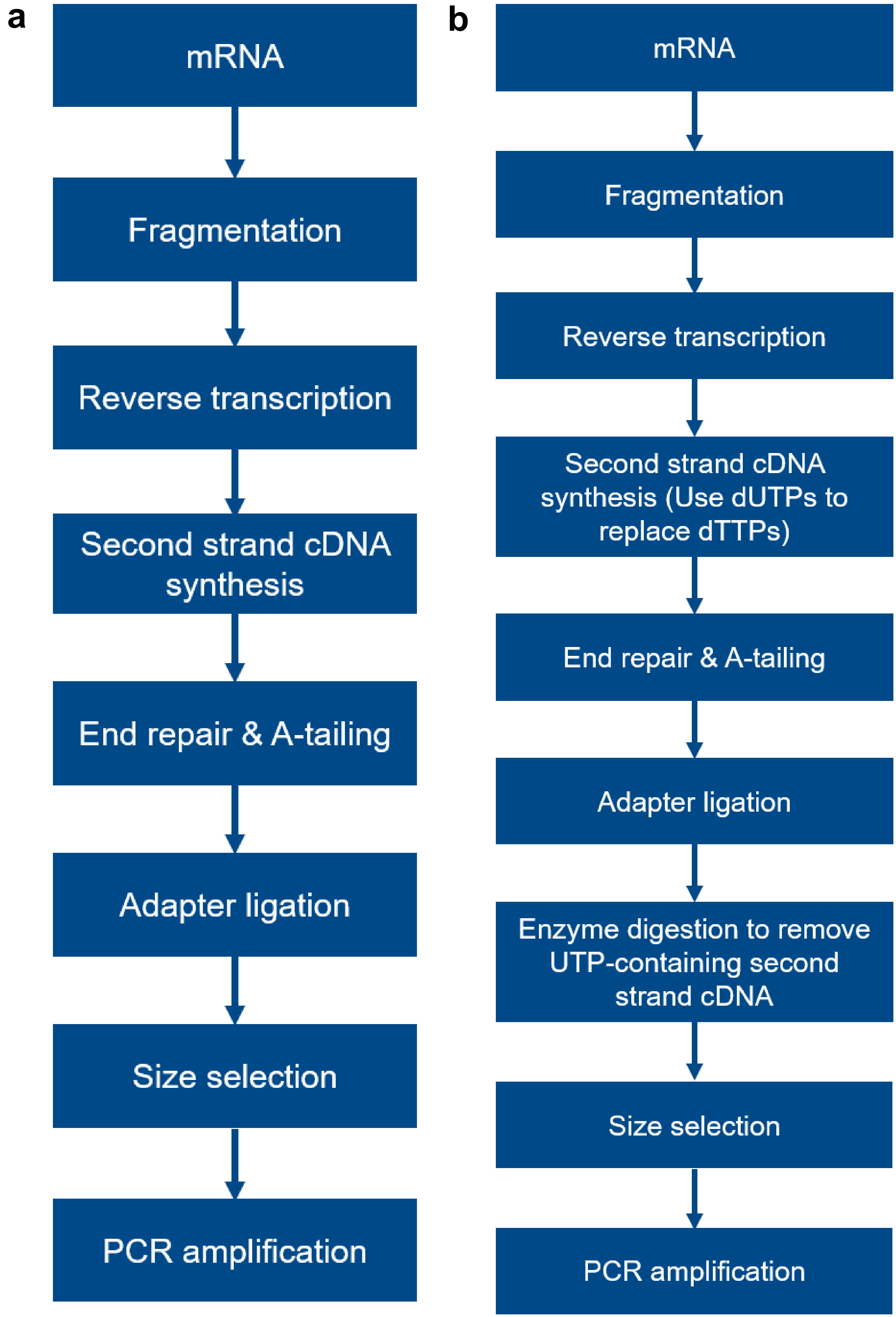

Supplementary Figure S2: Workflow for library preparation for mRNA sequencing of *cdc10-129* cells and wild type cells. a) Workflow of non-directional library construction. b) Workflow for directional library construction.

Supplementary Figure 3

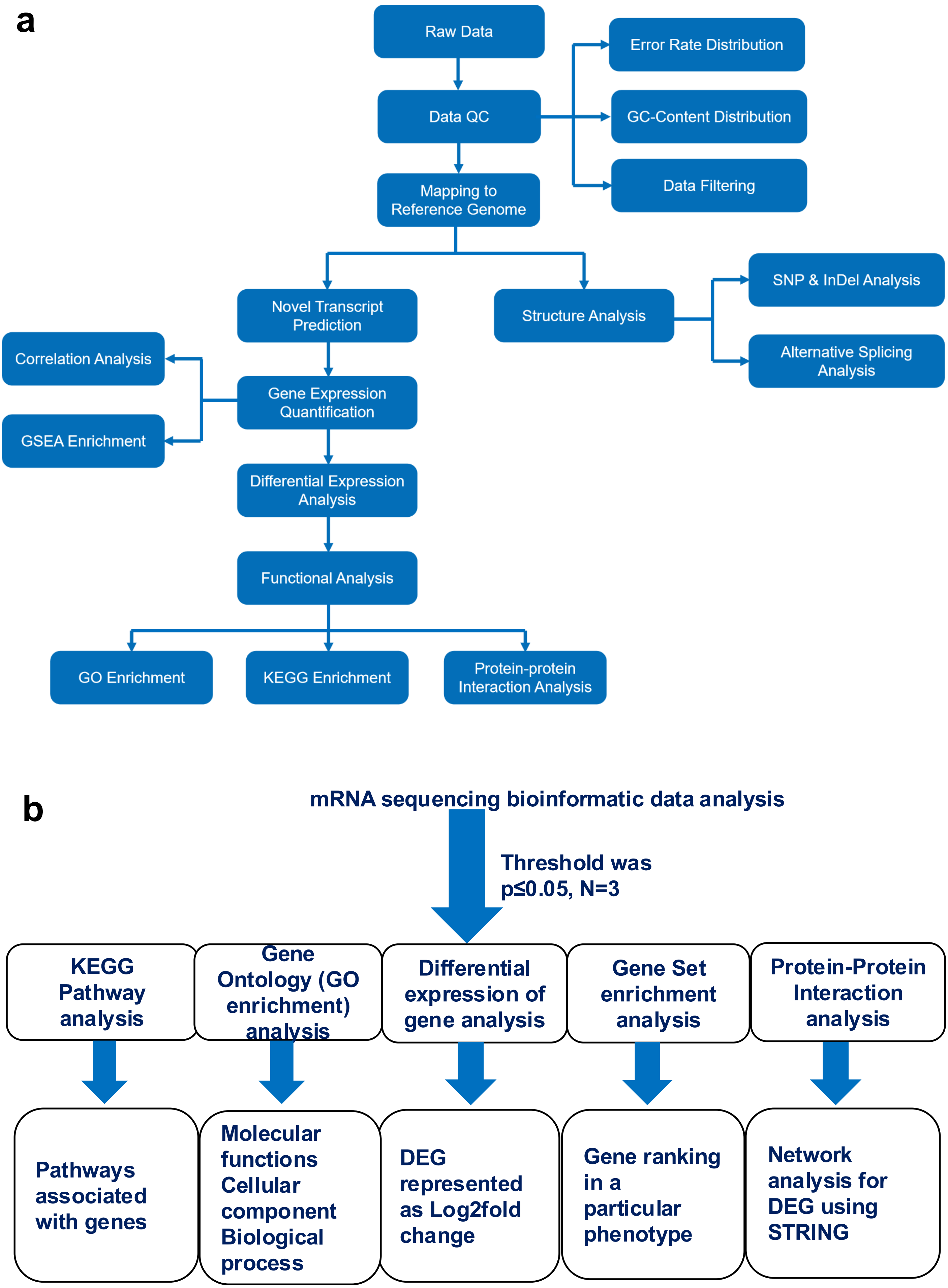

Supplementary Figure 3: Project workflow for a) mRNA sequencing information analysis technology flow. b) Data analysis for differential expression of genes, gene set from 3 independent biological replicates (N=3) with threshold of $p \leq 0.05$;

Supplementary Figure 4

a

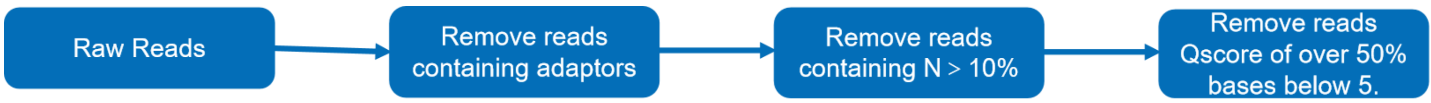

b

Adapter	Sequence
**P5 Adapter**	P5-AATGATACGGCGACCACCGAGA (5'-3')
	P5'-TTACTATGCCGCTGGTGGCTCT (3'-5')
**P7 Adapter**	CGTATGCCGTCTTCTGCTTG-P7' (5'-3')
	GCATACGGCAGAAGACGAAC-P7 (3'-5')

c

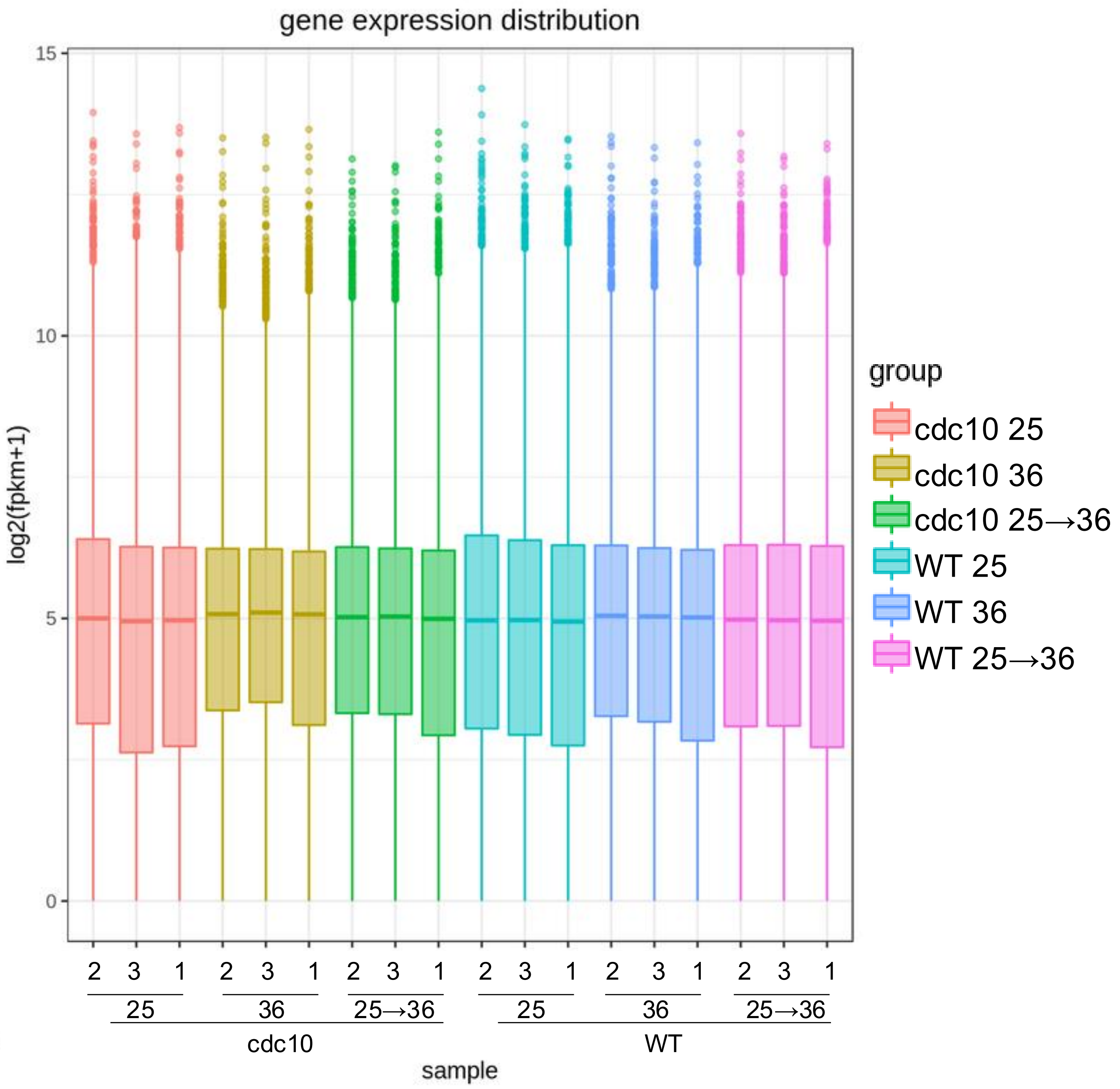

Supplementary Figure 4: Project workflow for: a) mRNA sequencing information analysis technology flow. b) Sequencing data filtration. B) Adapter sequence for the mRNA sequencing technology used in the high-throughput raw data collection. C) Sample gene expression distribution box plot. X axis represents the name of the sample, Y axis indicates the log2(FPKM+1), parameters of box plots are indicated, including maximum, upper quartile, mid-value, lower quartile and minimum.

Supplementary Figure 5

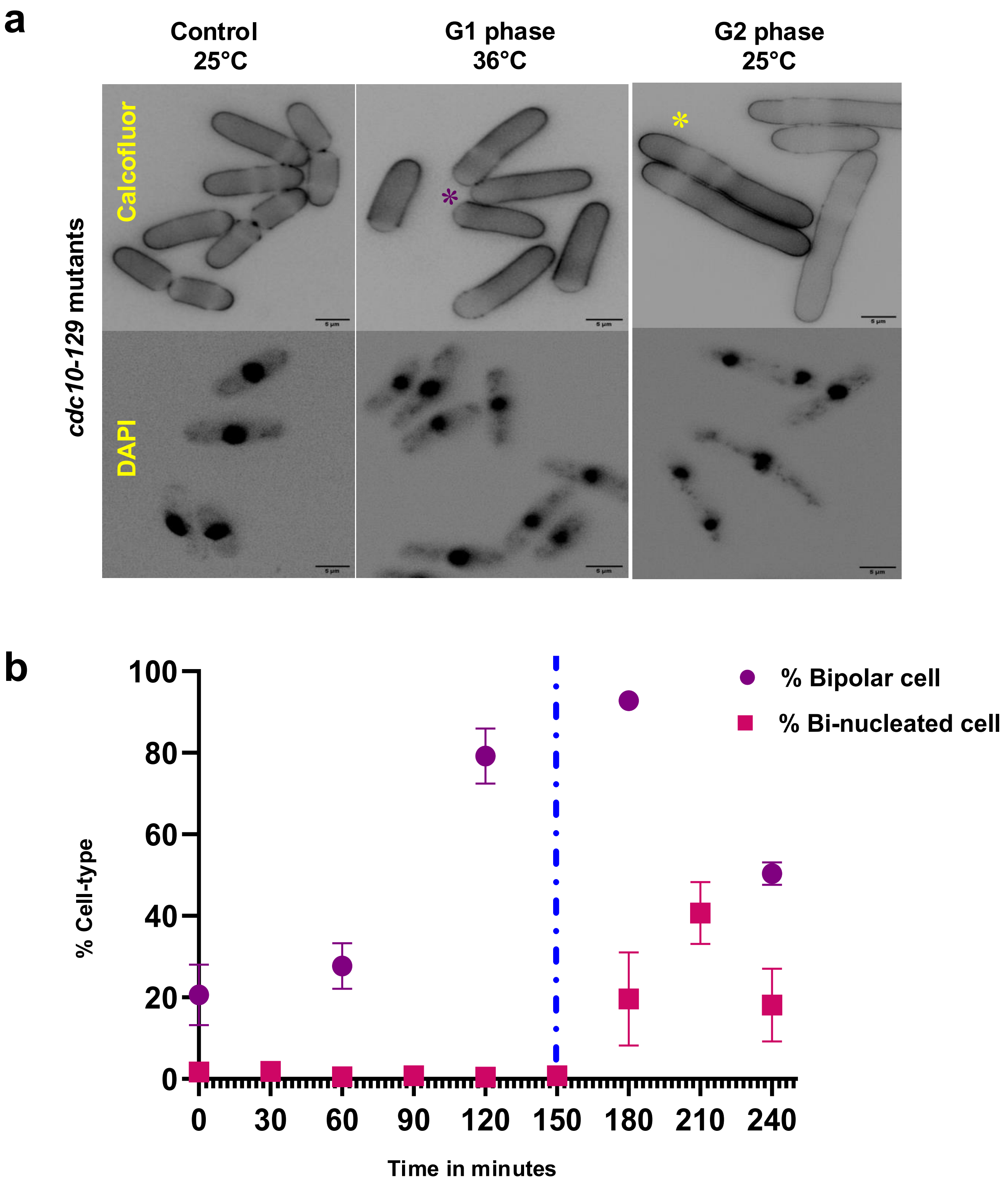

Supplementary Figure 5: Cell-cycle progression analysis of *cdc10-129* temperature-sensitive mutant cells post-release at 25°C using DAPI and Calcofluor staining: a) Calcofluor and DAPI stained cells as indicated. Purple asterisk mark monopolar cells and yellow asterisk mark bipolar cells. Scale bar= 5μm. b) Quantification of Time V/S Percentage cell-type post-release at 25°C. Purple dot represents the % bipolar cells whereas pink square represents the % bi-nucleated cells, error bar represents the standard deviation.

Supplementary Figure 6

a

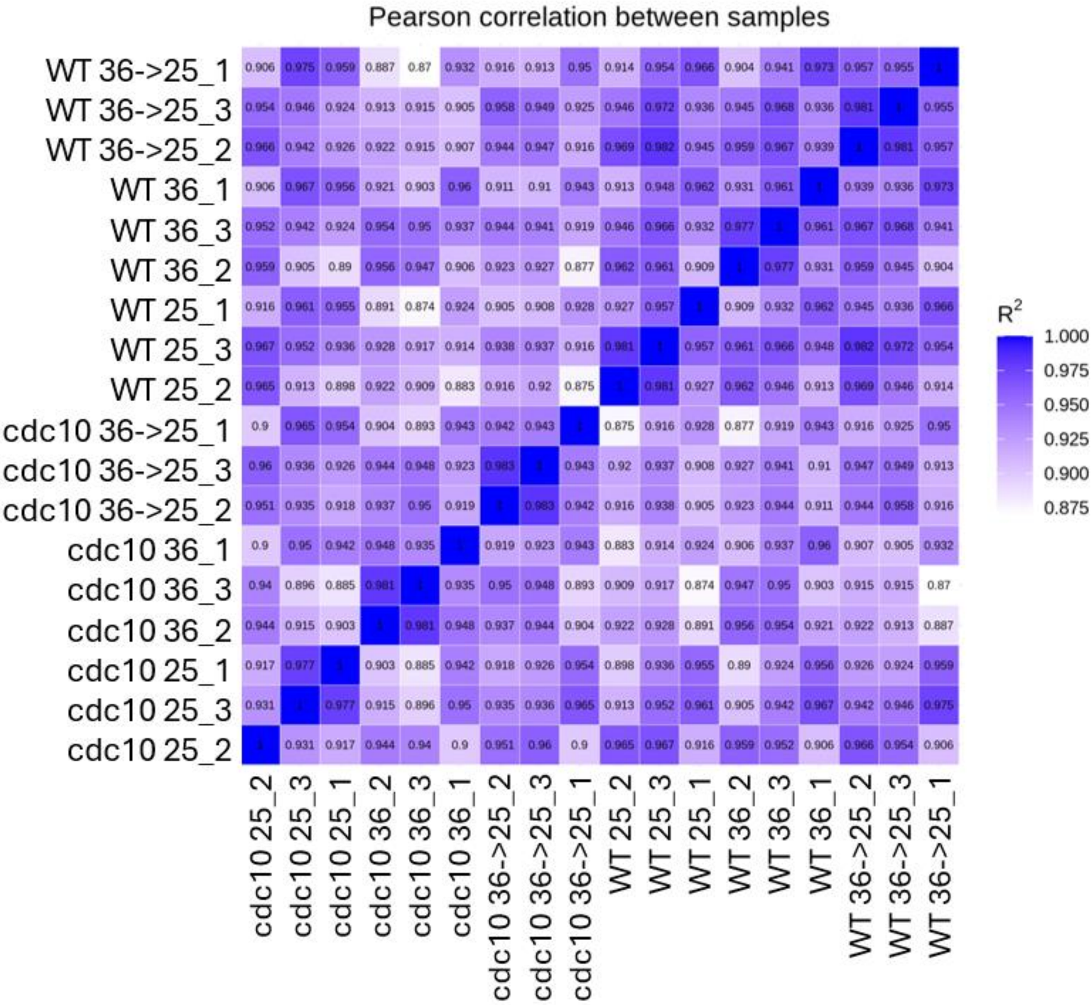

b

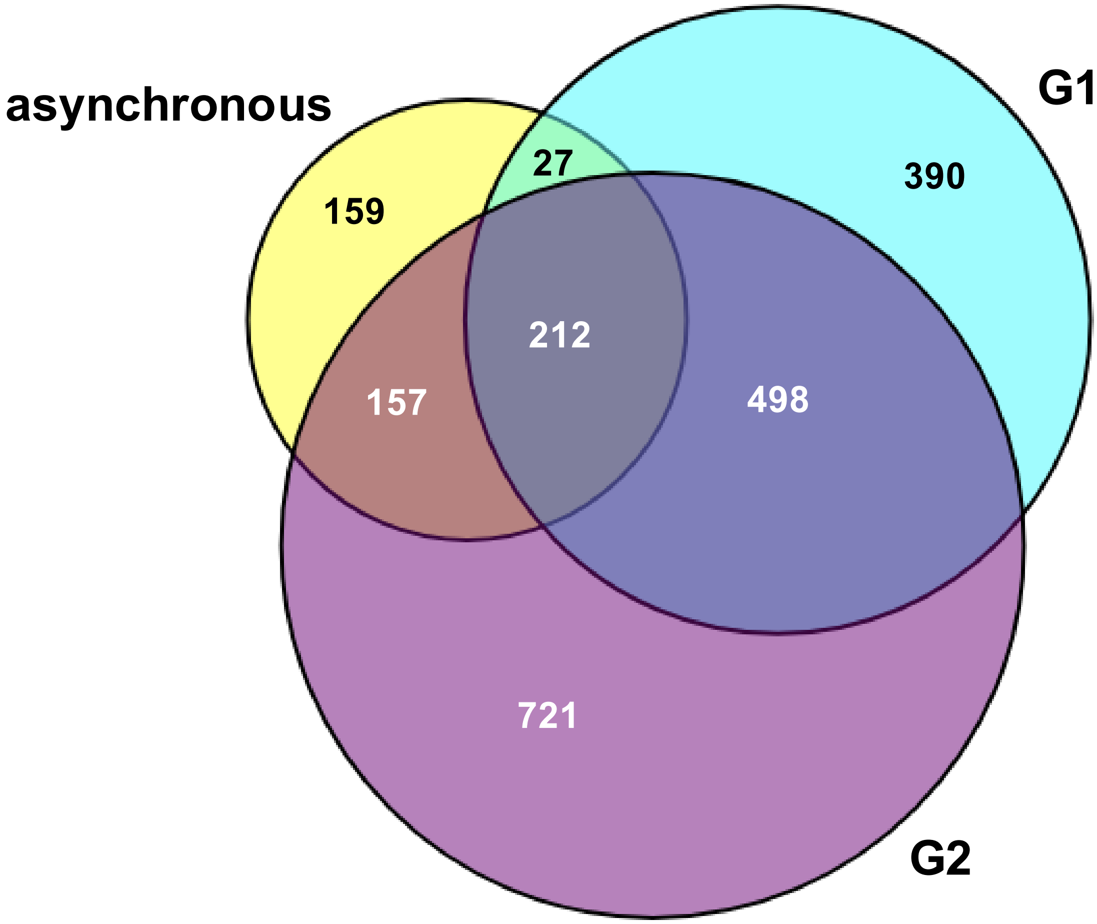

Supplementary Figure 6: Data quality control and analysis for mRNA sequencing. a) Inter-sample correlation heatmap where R2 is Square of Pearson correlation coefficient (R). b) Differential expression of gene represented as a Venn diagram with asynchronous *cdc10-129* cells at 25°C, synchronized G1 arrested *cdc10-129* at 36°C, and synchronized G2 phase cells post-release shifted from 36°C to 25°C for 150 minutes, after correction for temperature shift effects.

Supplementary Figure 7

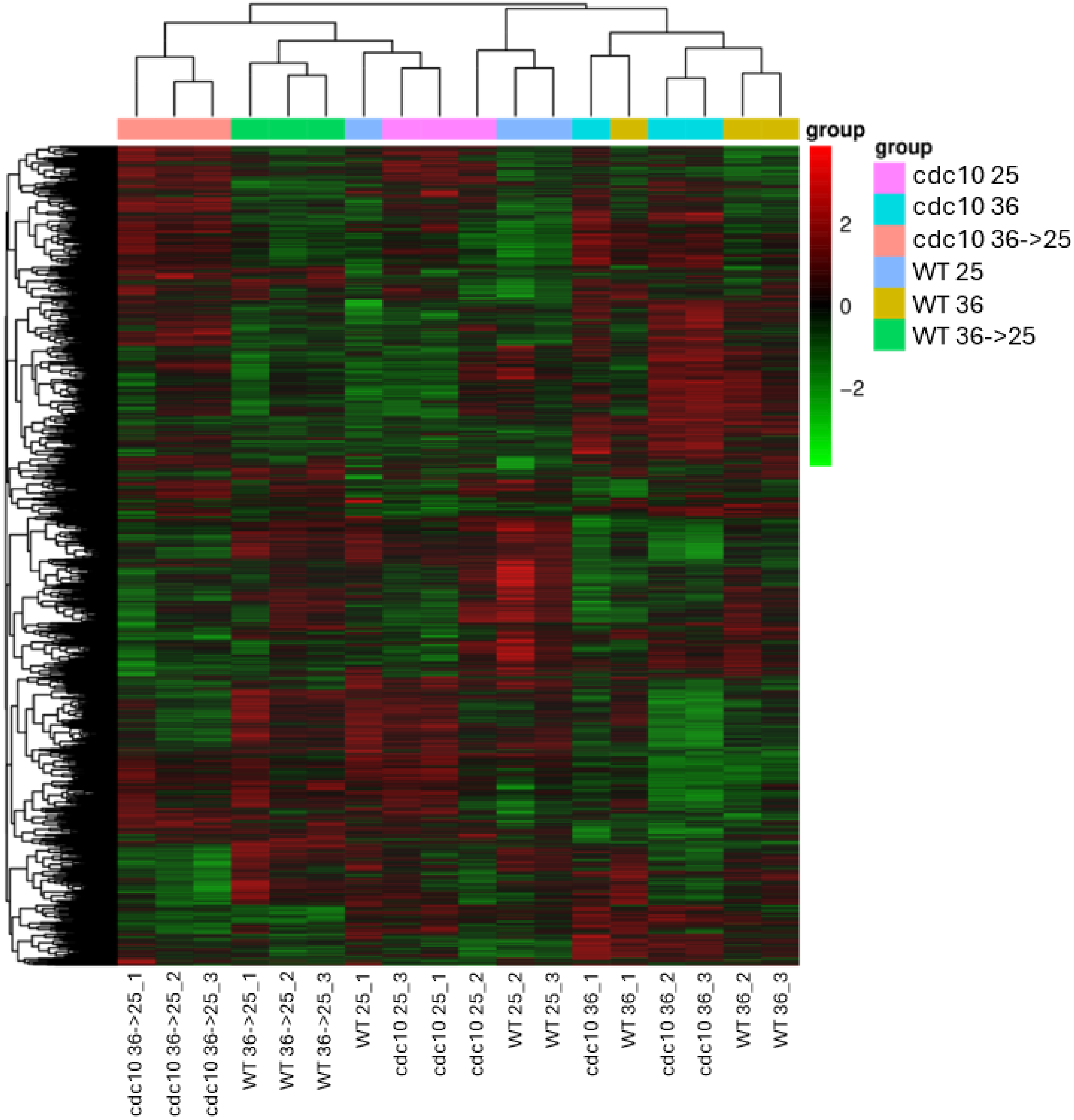

Supplementary Figure 7: Heatmap representation of differential gene clustering. The results of FPKM inter-group cluster analysis using the log2(FPKM+1) value. Red indicates genes with high expression levels, and green indicates genes with low expression levels.

Supplementary Figure 8

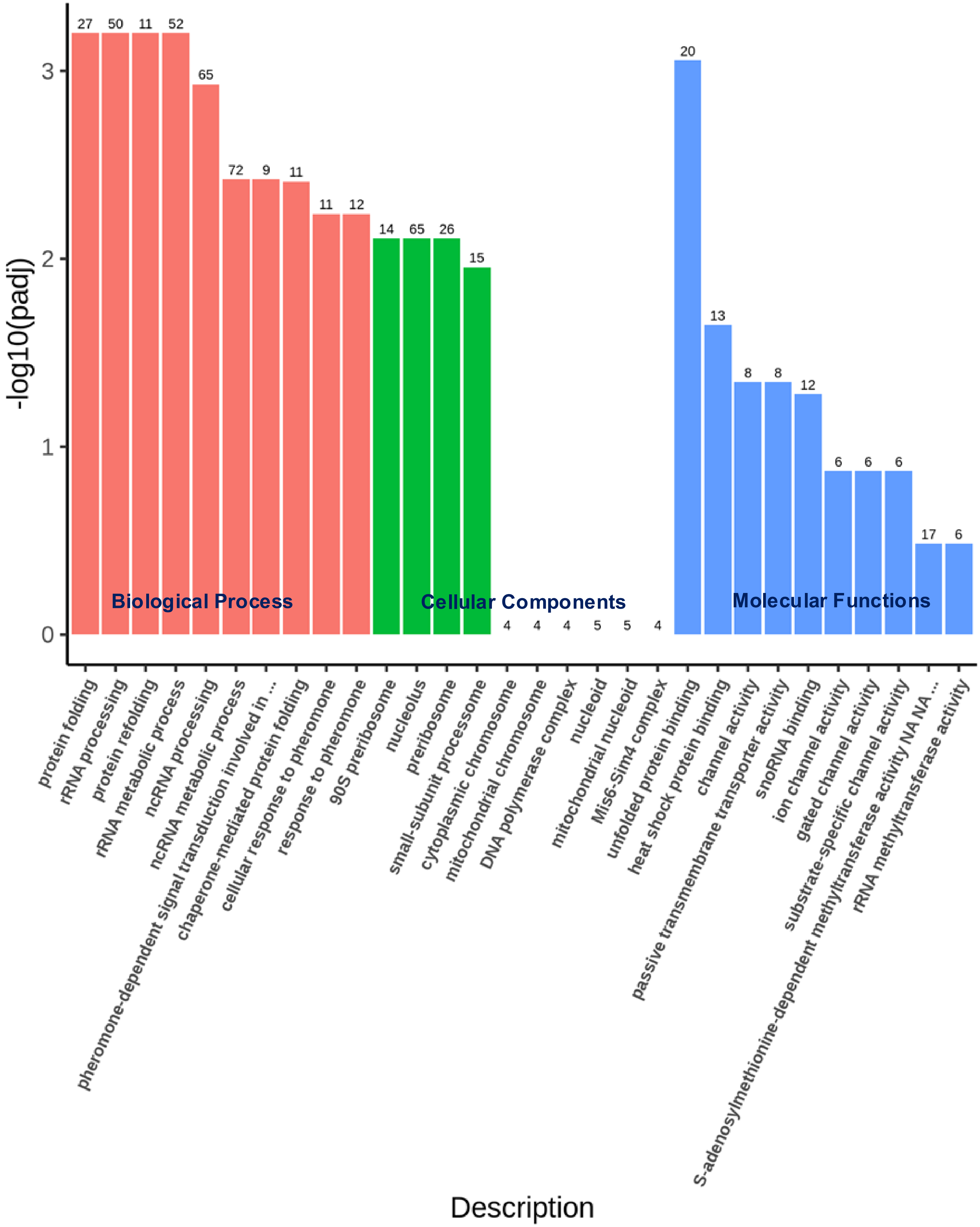

Supplementary Figure 8: GO enrichment histograms for genes upregulated in G1 phase cells. The abscissa in the figure shows GO Terms, and the ordinate is the level of significance of enrichment, expressed as -log10(padj). Different colors represent different functional categories**.** Orange for Biological Process, Green for Cellular Components and Blue for Molecular Functions.

Supplementary Figure 9

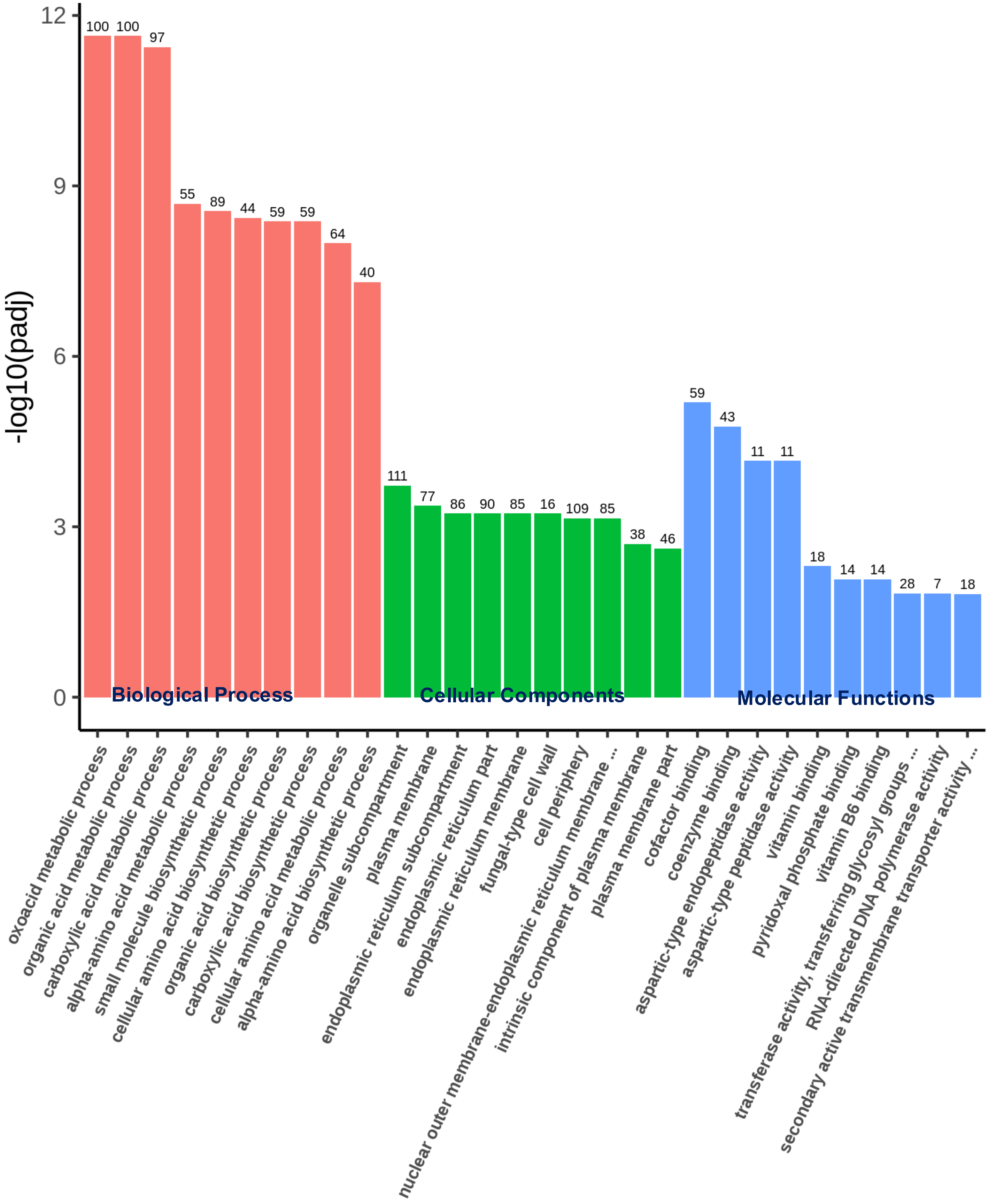

Supplementary Figure 9: GO enrichment histograms for genes upregulated in G2 phase cells. The abscissa in the figure shows GO Terms, and the ordinate is the level of significance of enrichment, expressed as -log10(padj). Different colors represent different functional categories**.** Orange for Biological Process, Green for Cellular components and Blue for Molecular Functions.

Supplementary Figure 10

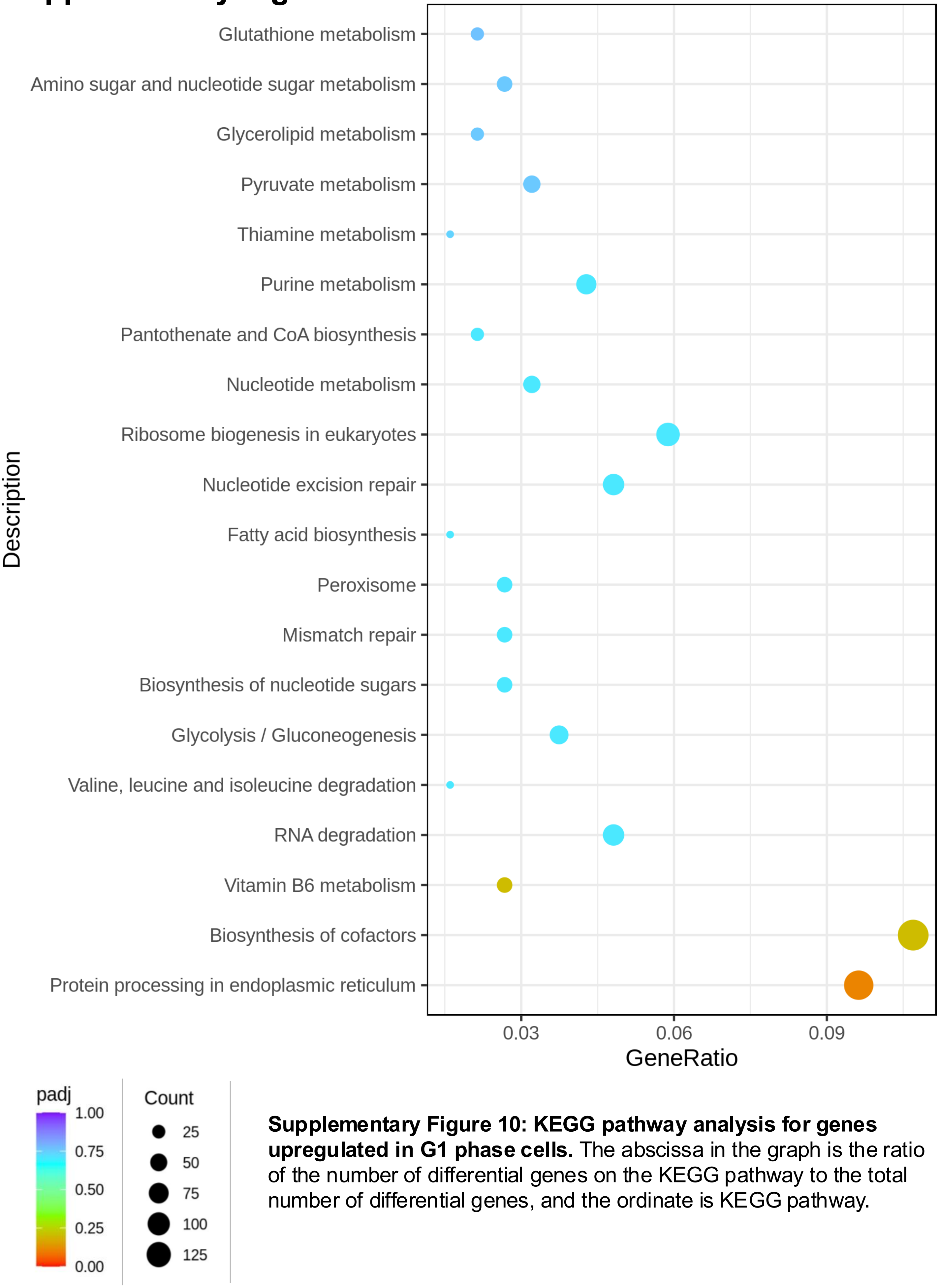

Supplementary Figure 10: KEGG pathway analysis for genes upregulated in G1 phase cells. The abscissa in the graph is the ratio of the number of differential genes on the KEGG pathway to the total number of differential genes, and the ordinate is KEGG pathway.

Supplementary Figure 11

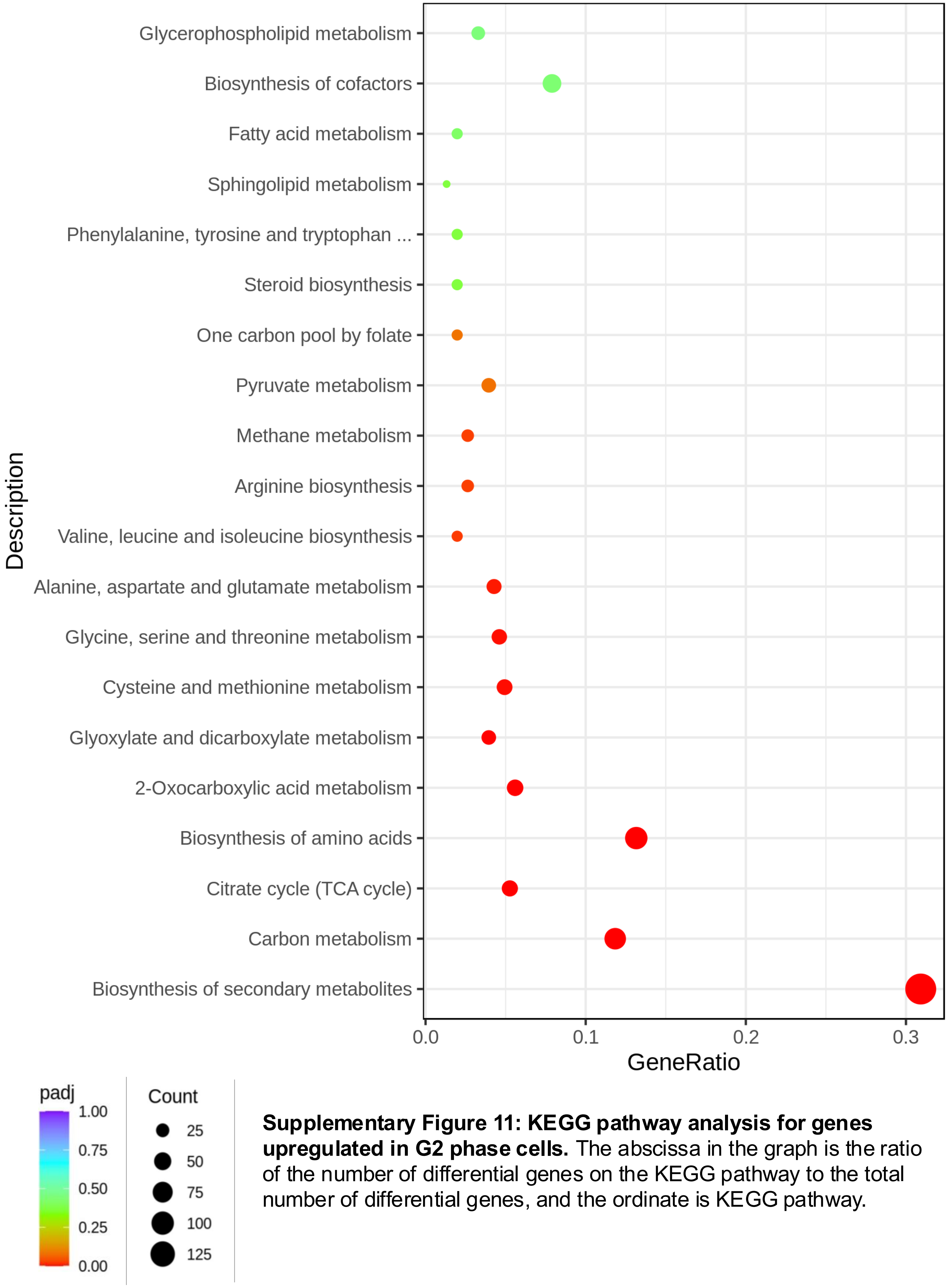

Supplementary Figure 11: KEGG pathway analysis for genes upregulated in G2 phase cells. The abscissa in the graph is the ratio of the number of differential genes on the KEGG pathway to the total number of differential genes, and the ordinate is KEGG pathway.

Supplementary Figure 12

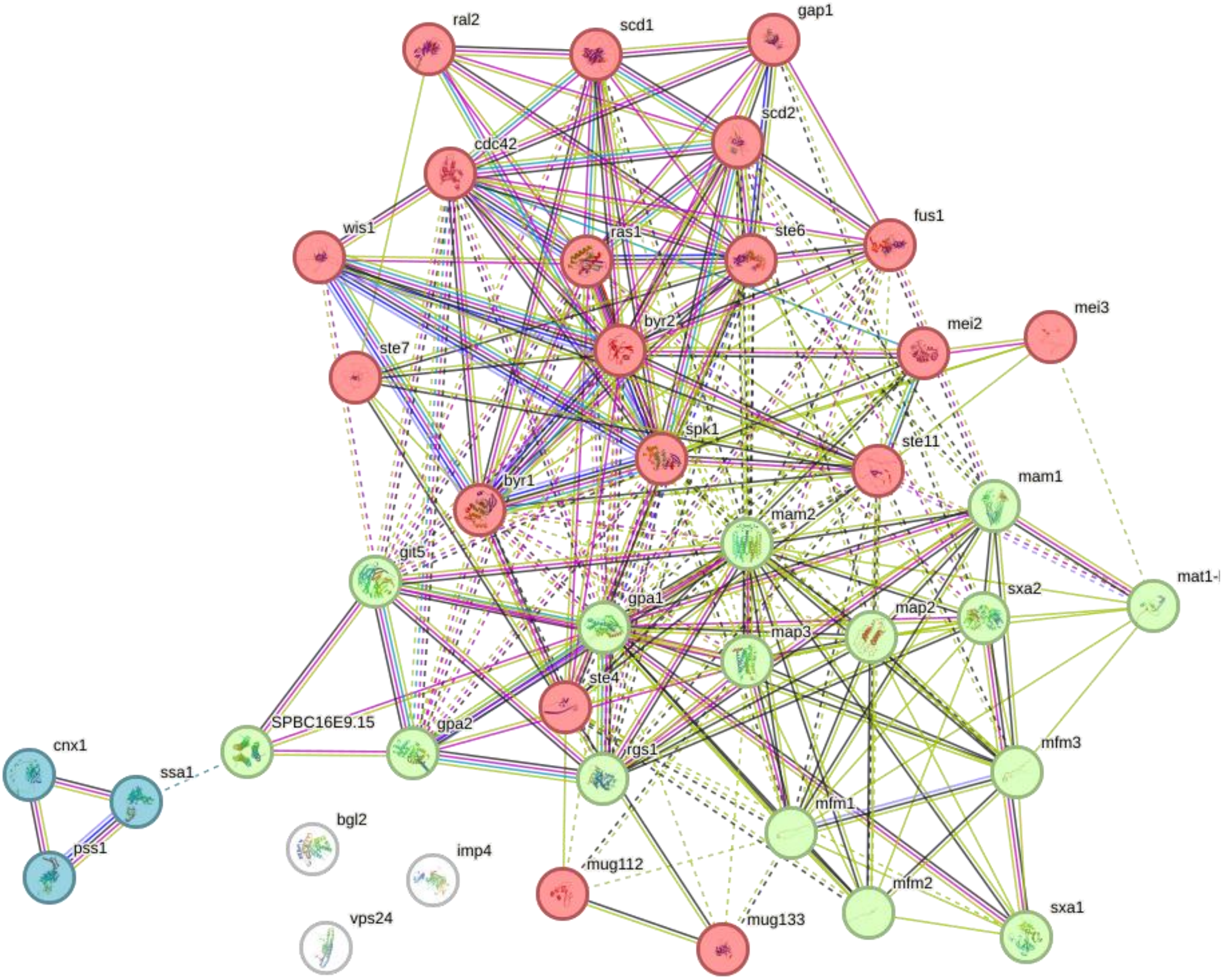

Supplementary Figure 12: Protein-protein interaction analysis using STRING for upregulated genes in the G1 phase of *cdc10-129* cells. Protein-protein interaction (PPI) network generated using STRING database showing genes upregulated in G1 phase. Input genes were mapped to the STRING database. Additional interacting proteins not present in the original input list were incorporated by STRING to extend the network. There are total of 40 nodes including 225 edges in the figure representing the upregulated genes in the G1 phase cells. The average node degree is estimated to be 11.2 whereas average local clustering co-efficient is 0.628. PPI enrichment value was estimated to be < 1.0e-16. Three basic cluster appears in the PPI analysis using k-means clustering.
Red outline around the genes indicate genes involved in positive regulation of conjugation in cellular fusion. Green outline around the genes indicate genes involved in signal transduction and positive regulation of conjugation with cellular fusion and signaling receptor binding. Blue outline around the genes indicate genes involved in heat shock protein related to protein folding pathways. Grey outlines do not belong interact with the main clusters. Edges represent protein-protein interactions, with solid lines indicating interactions supported by "direct" or high-confidence evidence while dashed lines indicate predicted or indirect interactions.

Supplementary Figure 13

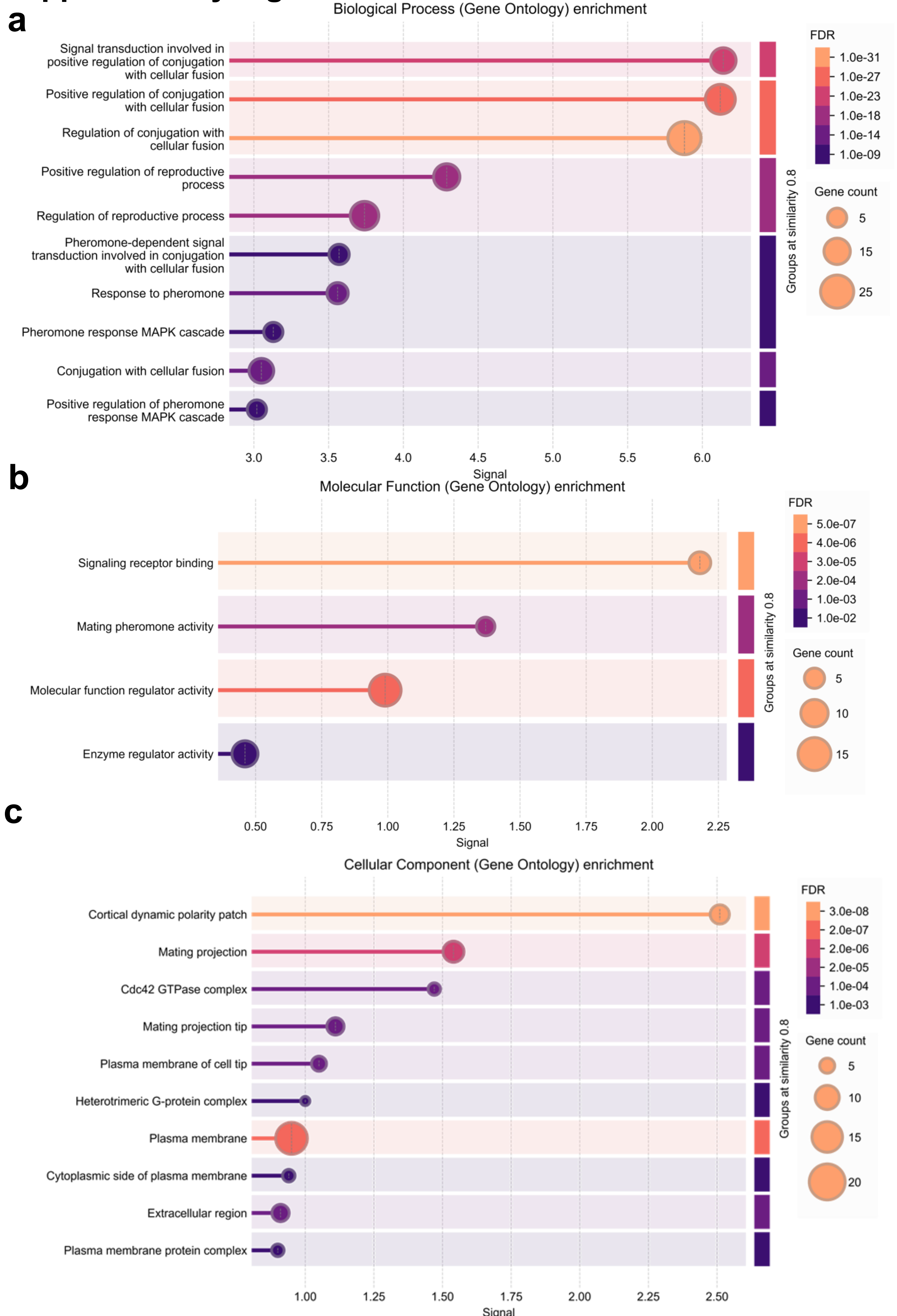

Supplementary Figure 13: Protein-protein interaction analysis using STRING for upregulated genes in the G1 phase of *cdc10-129* cells. a) Biological Process (Gene Ontology) enrichment pathways (top 10 pathways) b) Molecular Functions (Gene Ontology) enrichment pathways (top 4 pathways) c) Cellular Component (Gene Ontology) enrichment pathways (top 10 pathways).

Supplementary Figure 14

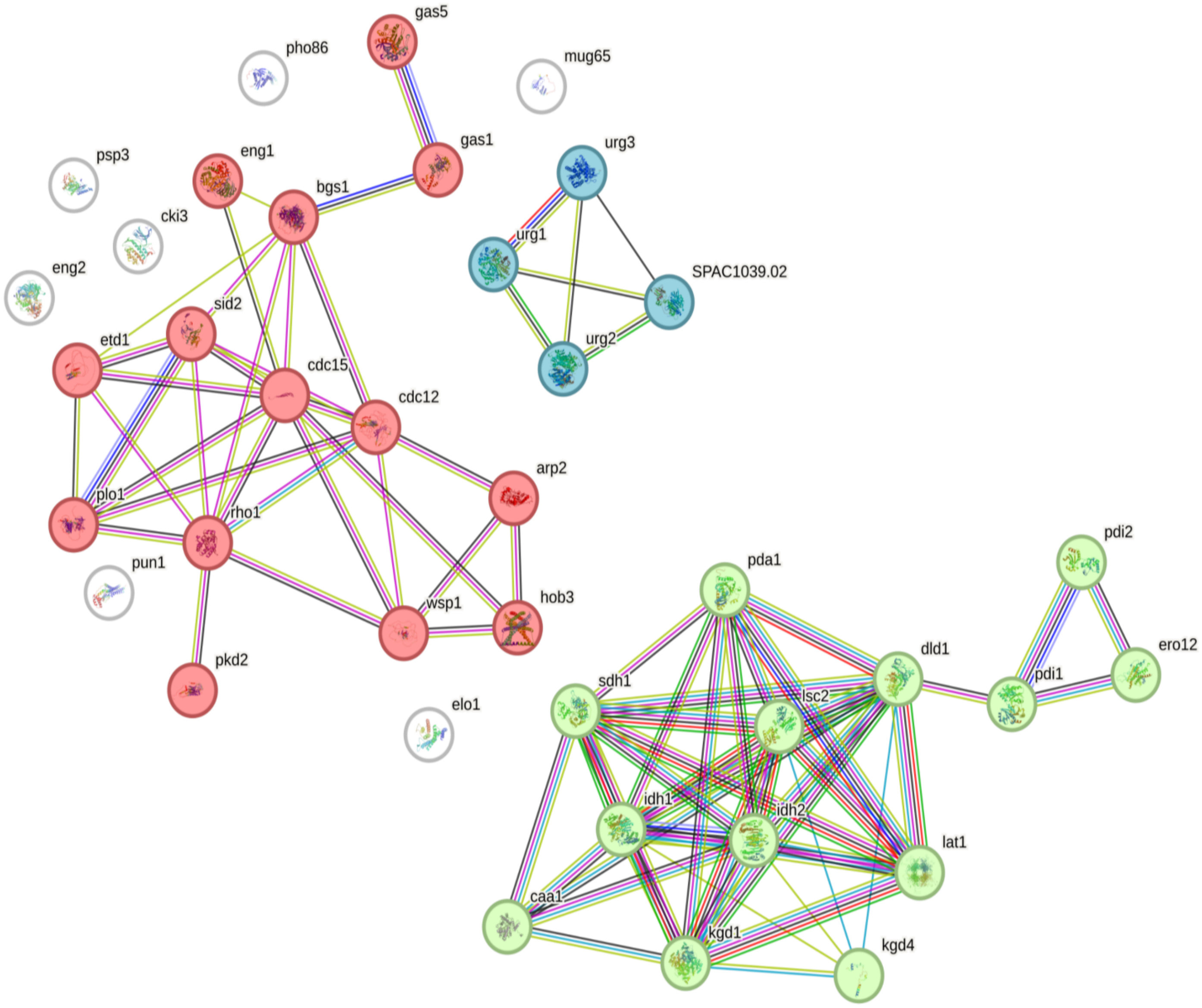

Supplementary Figure 14: Protein-protein interaction analysis of the downregulated genes in the G1 phase of *cdc10-129* cells using STRING analysis. Protein-protein interaction (PPI) network generated using STRING database showing genes down regulated in G1 phase. Input genes were mapped to the STRING database. Additional interacting proteins not present in the original input list were incorporated by STRING to extend the network. There are total of 38 nodes including 80 edges in the figure representing the down-regulated genes in the G1 phase cells. The average node degree was estimated to be 4.21 whereas average local clustering co-efficient is 0.654. PPI enrichment value was estimated to be <1.0e-16. Three basic cluster appears in the PPI analysis using k-means clustering. Red outline around the genes indicate genes involved in positive regulation of cellular component biogenesis. Green outline around the genes indicate genes involved in the citric acid cycle (TCA cycle). Blue outline around the genes indicate genes involved in mixed pathways including uracil phosphoribosyltransferase, and regulation of pyrimidine-containing compound salvage. Grey outlines do not interact with the main clusters. Edges represent protein-protein interactions, with solid lines indicating interactions supported by "direct" or high-confidence evidence while dashed lines indicate predicted or indirect interactions.

Supplementary Figure 15

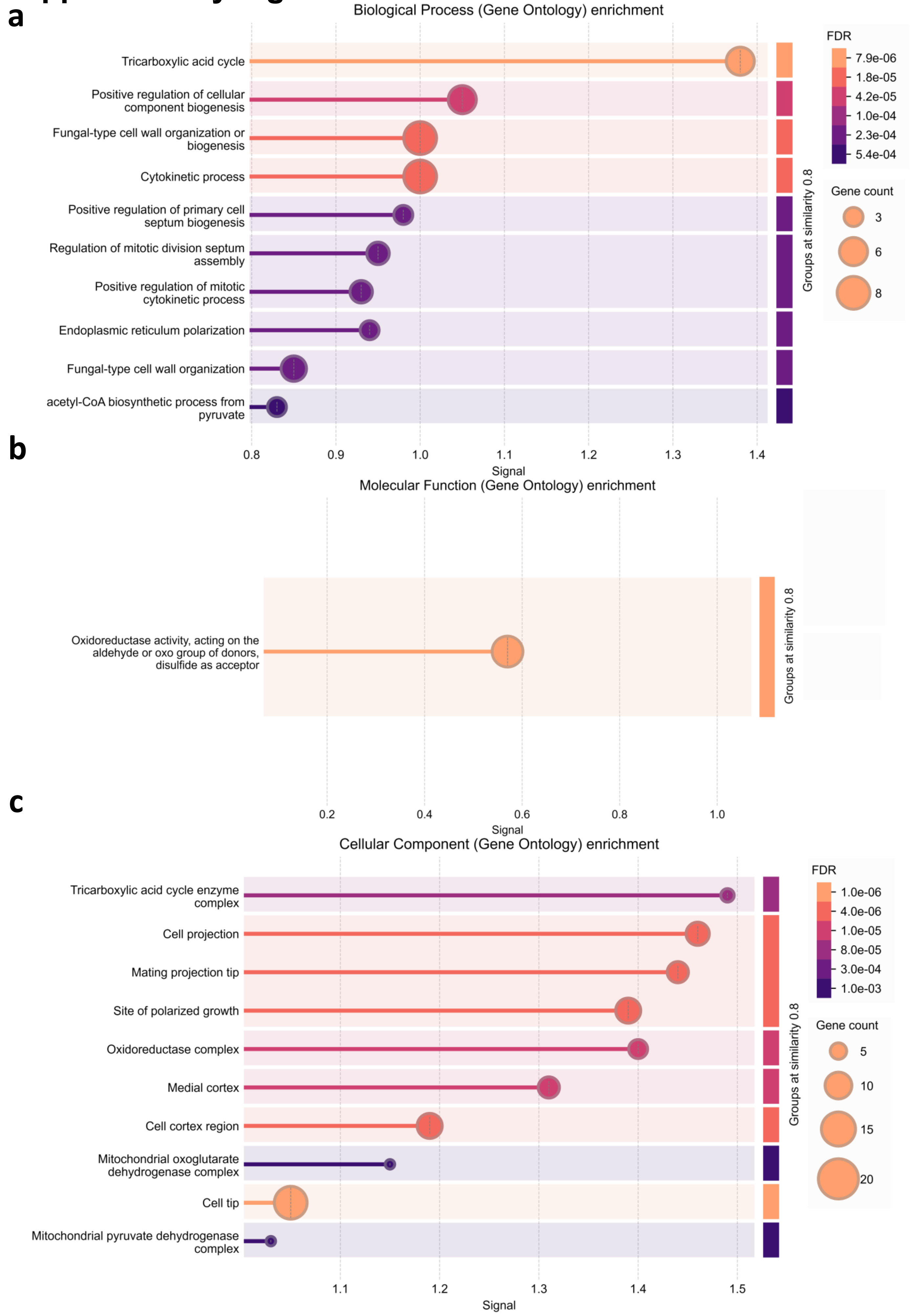

Supplementary Figure 15: Protein-protein interaction analysis using STRING for downregulated genes in the G1 phase of *cdc10-129* cells. a) Biological Process (Gene Ontology) enrichment pathways (top 10 pathways) b) Molecular Functions (Gene Ontology) enrichment pathways (top 1 pathways) c) Cellular Component (Gene Ontology) enrichment pathways (top 10 pathways).

Supplementary Figure 16

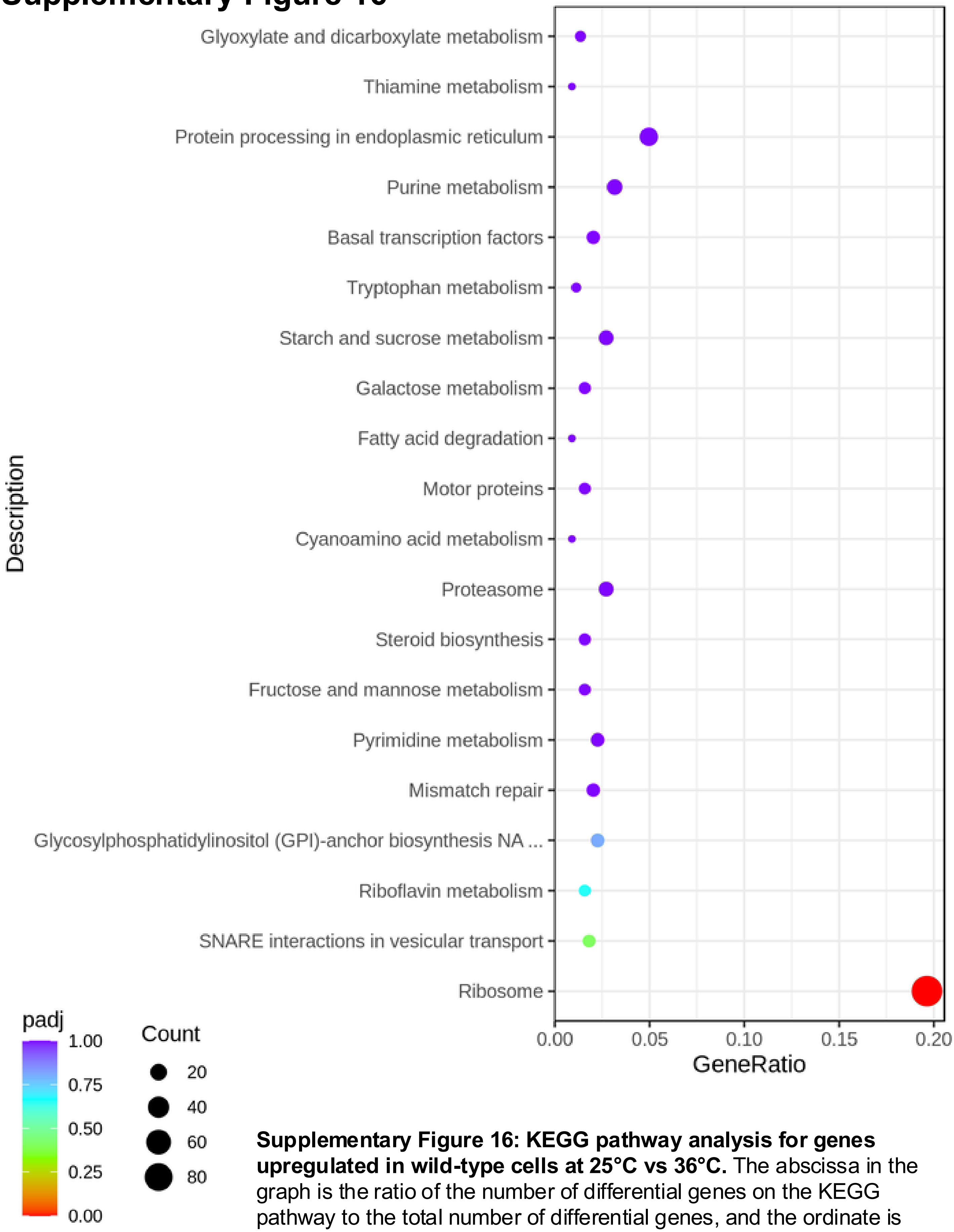

Supplementary Figure 16: KEGG pathway analysis for genes upregulated in wild-type cells at 25°C vs 36°C. The abscissa in the graph is the ratio of the number of differential genes on the KEGG pathway to the total number of differential genes, and the ordinate is KEGG pathway.
